# Metalloprotease inhibitor TIMP proteins control FGF-2 bioavailability and regulate skeletal growth

**DOI:** 10.1083/jcb.201906059

**Published:** 2019-08-01

**Authors:** Sanjay Saw, Alison Aiken, Hui Fang, Trevor D. McKee, Sarah Bregant, Otto Sanchez, Yan Chen, Ashley Weiss, Brendan C. Dickson, Bertrand Czarny, Ankit Sinha, Amanda Fosang, Vincent Dive, Paul D. Waterhouse, Thomas Kislinger, Rama Khokha

**Affiliations:** 1 Princess Margaret Cancer Centre/Ontario Cancer Institute, University Health Network, Toronto, Canada; 2 Institute of Biology and Technology, Saclay, France; 3 University of Ontario Institute of Technology, Oshawa, Canada; 4 Mount Sinai Hospital, Toronto, Canada; 5 University of Melbourne Department of Paediatrics and Murdoch Children’s Research Institute, Royal Children’s Hospital, Parkville, Victoria, Australia

## Abstract

Regulated growth plate activity is essential for postnatal bone development and body stature, yet the systems regulating epiphyseal fusion are poorly understood. Here, we show that the tissue inhibitors of metalloprotease (TIMP) gene family is essential for normal bone growth after birth. Whole-body quadruple-knockout mice lacking all four TIMPs have growth plate closure in long bones, precipitating limb shortening, epiphyseal distortion, and widespread chondrodysplasia. We identify TIMP/FGF-2/IHH as a novel nexus underlying bone lengthening where TIMPs negatively regulate the release of FGF-2 from chondrocytes to allow IHH expression. Using a knock-in approach that combines MMP-resistant or ADAMTS-resistant aggrecans with TIMP deficiency, we uncouple growth plate activity in axial and appendicular bones. Thus, natural metalloprotease inhibitors are crucial regulators of chondrocyte maturation program, growth plate integrity, and skeletal proportionality. Furthermore, individual and combinatorial TIMP-deficient mice demonstrate the redundancy of metalloprotease inhibitor function in embryonic and postnatal development.

## Abstract (precis)

Saw et al. show via the combinatorial deletion of Timp family members in mice that metalloprotease regulation of FGF-2 is a crucial event in the chondrocyte maturation program, underlying the growth plate development and bone elongation responsible for attaining proper body stature.

## Introduction

Metalloproteases are present across all kingdoms of living organisms and have expanded widely during eukaryotic evolution, comprising the largest class of protease genes in humans (Gomis-Rüth, 2003; Quesada et al., 2009). Tissue inhibitors of metalloproteases (TIMPs) are well known to control the turnover of matrix proteins in connective tissue (Sterchi et al., 2008). The TIMP gene family has been diversified with phylogenic development. Flies have a single TIMP gene with conserved metalloprotease inhibitor function, loss of which causes blistered wings and death by digestive tract lysis (Godenschwege et al., 2000; Page-McCaw et al., 2003). The five putative TIMPs in zebrafish have not been well studied (Wyatt et al., 2009). Mammals possess four TIMPs, which inhibit most metzincins, a subfamily of 89 secreted and cell surface–bound metalloproteases (Sterchi et al., 2008). Beyond matrix turnover, the TIMP-metalloprotease axis controls major signaling pathways through ectodomain shedding, and deregulation of this axis has invariably been seen in human cancers and diseases (Murphy et al., 2008; Aiken and Khokha, 2010; Kessenbrock et al., 2010; Jackson et al., 2017). Overlapping enzyme inhibitory specificity among TIMP proteins undermines the ability of single TIMP knockout mice to reveal their critical biology. Therefore, concurrent deletion of the entire TIMP gene family is a prerequisite for understanding the function of this gene family in mammals.

Postnatal bone growth employs isometric scaling (Pietak et al., 2013; Stern et al., 2015), and skeletal proportionality varies in primates, where it has evolved to provide optimal biomechanical efficiency for species-specific adaptation to their habitat. In humans, the combined length of femur plus tibia is ∼50% of total stature, which is biomechanically efficient for a striding bipedal gait (Bogin and Varela-Silva, 2010). Body stature relies on height attained during the growth of long bones. Endochondral ossification is the sole process of bone elongation and is accomplished through the replacement of cartilage by bone matrix underneath the growth plate. Mesenchymal cells in the growth plate differentiate into chondrocytes, sequentially generating proliferating and hypertrophic zones, with the hypertrophic chondrocytes ultimately undergoing apoptosis to leave behind a cartilage matrix for subsequent ossification. Long bones continue to grow until growth plate closure with sexual maturity postpuberty. This defined chondrocyte program of proliferation, differentiation, and maturation is tightly regulated by local factors. The morphogen Indian hedgehog (IHH) and its interplay with parathyroid hormone–related protein are important to balance proliferating and hypertrophic chondrocytes (Kobayashi et al., 2002; Mizuhashi et al., 2018; Newton et al., 2019). Signaling from fibroblast growth factor receptor 3 (FGFR3) antagonizes *Ihh* expression in chondrocytes and leads to alteration in growth plate activity (Minina et al., 2002; Tang et al., 2016). Extracellular matrix proteins are also important regulators of growth plate activity (Aiken and Khokha, 2010). Aggrecan is a signatory proteoglycan of chondrocyte matrix and mutations in this molecule underlie various chondrodysplasias in humans and other animals. Metalloproteases are essential enzymes for aggrecan turnover, and endogenous TIMPs keep the protease activity in check. TIMPs are expressed in most organs including bone and cartilage, and their deregulation has been reported in cartilage and bone pathologies (Gendron et al., 2003; Nuttall et al., 2004; Aiken and Khokha, 2010; Chen et al., 2019).

Here we characterize cartilage in genetically engineered mouse models (GEMMs) lacking all four TIMP genes. Complete TIMP deficiency produces severe skeletal defects due to an aberrant chondrocyte maturation program during the major period of bone growth spanning birth to puberty. We identify a TIMP-dependent regulation of FGF-2 and IHH signaling in the growth plate. Using compound GEMMs, we rescue the bone defects by incorporation of aggrecan knock-in mutations resistant to either matrix metalloproteinase (MMP) or ADAMTS cleavage, illustrating the requirement of TIMP-regulated metalloprotease activity for correct bone proportionality. The phenotypic manifestations in the TIMPless mice provide fundamental insights into the molecular drivers of mammalian skeletal growth and stature, as well as expose the functional redundancy in the metalloprotease inhibitor gene family.

## Results

### Abnormal bone growth and isometry in quadruple TIMP-deficient mice

**Figure 1.**
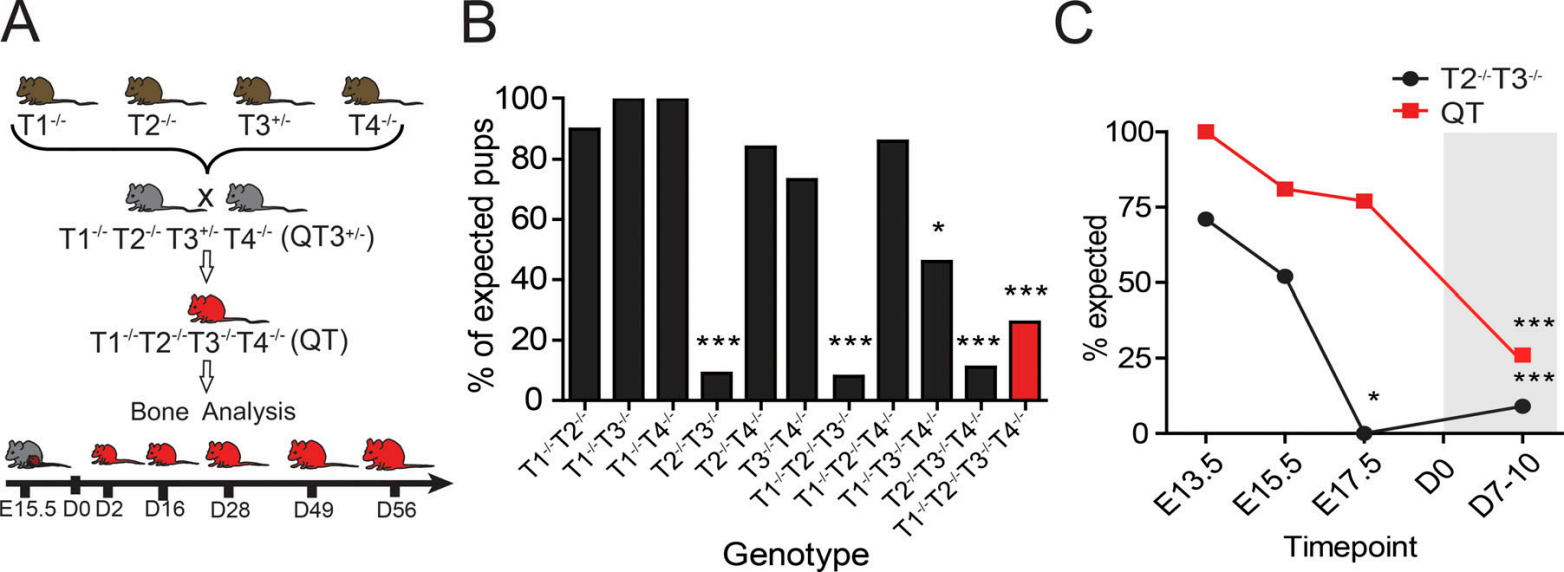
**TIMP redundancy for in utero survivability. (A)** Breeding strategy for the generation of QT3^+/−^ and QT mice. Individual TIMP knockout mice were bred through several crosses to generate QT3^+/−^ mice. QT3^+/−^ were used as breeders to generate QT and QT3^+/−^ mice. **(B)** Combinations of compound TIMP knockout mice were bred, and pups were genotyped. Number of pups genotyped at 1 wk after birth: T1^−/−^ T2^−/−^, *n* = 60; T1^−/−^ T3^−/−^, *n* = 45; T1^−/−^ T4^−/−^, *n* = 103; T2^−/−^ T3^−/−^, *n* = 590; T2^−/−^ T4^−/−^, *n* = 80; T3^−/−^ T4^−/−^, *n* = 85; T1^−/−^ T2^−/−^ T3^−/−^, *n* = 252; T1^−/−^ T2^−/−^ T4^−/−^, *n* = 14; T1^−/−^ T3^−/−^ T4^−/−^, *n* = 58; T2^−/−^ T3^−/−^ T4^−/−^, *n* = 137; T1^−/−^ T2^−/−^ T3^−/−^ T4^−/−^, *n* = 473. χ^2^ test compared observed versus expected ratios for all genotypes. Comparison of expected (Mendelian distribution) versus observed revealed that the T2^−/−^T3^−/−^ combination results in near-complete in utero lethality (9 observed of 98 expected from 590 born pups). Other non-Mendelian genotypes were T1^−/−^ T2^−/−^ T3^−/−^ (3 of 37 from 252 born), T2^−/−^ T3^−/−^ T4^−/−^ (3 of 26 from 137 born), and T1^−/−^ T2^−/−^ T3^−/−^ T4^−/−^ (31 of 118 from 473 born). **(C)** Embryos were genotyped and observed versus expected ratios compared using the χ^2^ test. Gray shading distinguishes postnatal time points. Enumeration of embryos (E13.5, E15.7, and E17.5) and born offspring showed that most T2^−/−^ T3^−/−^ die by E17.5, in contrast to QT, which were observed at >75% of the expected number. Therefore, the loss of TIMP2 and TIMP3 is detrimental at late gestation, while further additive loss of both TIMP1 and TIMP4 rescues lethality. It is conceivable that networks causing lethality in the T2^−/−^ T3^−/−^ scenario are either bypassed or opposed by the new milieu generated by complete TIMP loss. Numbers examined at E13.5, T2^−/−^ T3^−/−^ (*n* = 17) and T1^−/−^ T2^−/−^ T3^−/−^ T4^−/−^ (*n* = 13); E15.5, T2^−/−^ T3^−/−^ (*n* = 31) and T1^−/−^ T2^−/−^ T3^−/−^ T4^−/−^ (*n* = 81); E17.5, T2^−/−^ T3^−/−^ (*n* = 43), T1^−/−^ T2^−/−^ T3^−/−^ T4^−/−^ (*n* = 58); postnatal, T2^−/−^ T3^−/−^ (*n* = 33) and T1^−/−^ T2^−/−^ T3^−/−^ T4^−/−^ (*n* = 473). *, P < 0.05; ***, P < 0.001.

Individual whole-body TIMP1–TIMP4 knockout mice are viable and display mild phenotypes (Soloway et al., 1996; Caterina et al., 2000; Wang et al., 2000; Leco et al., 2001; Koskivirta et al., 2010). The four TIMP-null mouse strains were crossed to generate double and triple knockouts. For quadruple knockouts (QT; T1^−/−^ T2^−/−^ T3^−/−^ T4^−/−^), we used breeders that lacked *Timp1*, *Timp2*, and *Timp4* and were *Timp3* heterozygous (QT3^+/−^; T1^−/−^ T2^−/−^ T3^+/−^ T4^−/−^; Fig. 1 A), as described in Materials and methods. Quadruple TIMP-deficient mice were born at a lower rate than expected (Fig. 1 B), were slow to gain weight after birth, and rarely survived past 8 wk. Surprisingly, a single *Timp3* allele in QT3^+/−^ mice was sufficient for a natural lifespan, whereas the complete absence of TIMP was incompatible with normal life expectancy. Furthermore, the combined loss of TIMP2 and TIMP3 unexpectedly resulted in embryonic lethality at late gestation, while the further additive loss of TIMP1 and TIMP4 rescued this lethality (Fig. 1, B and C).

**Figure 2.**
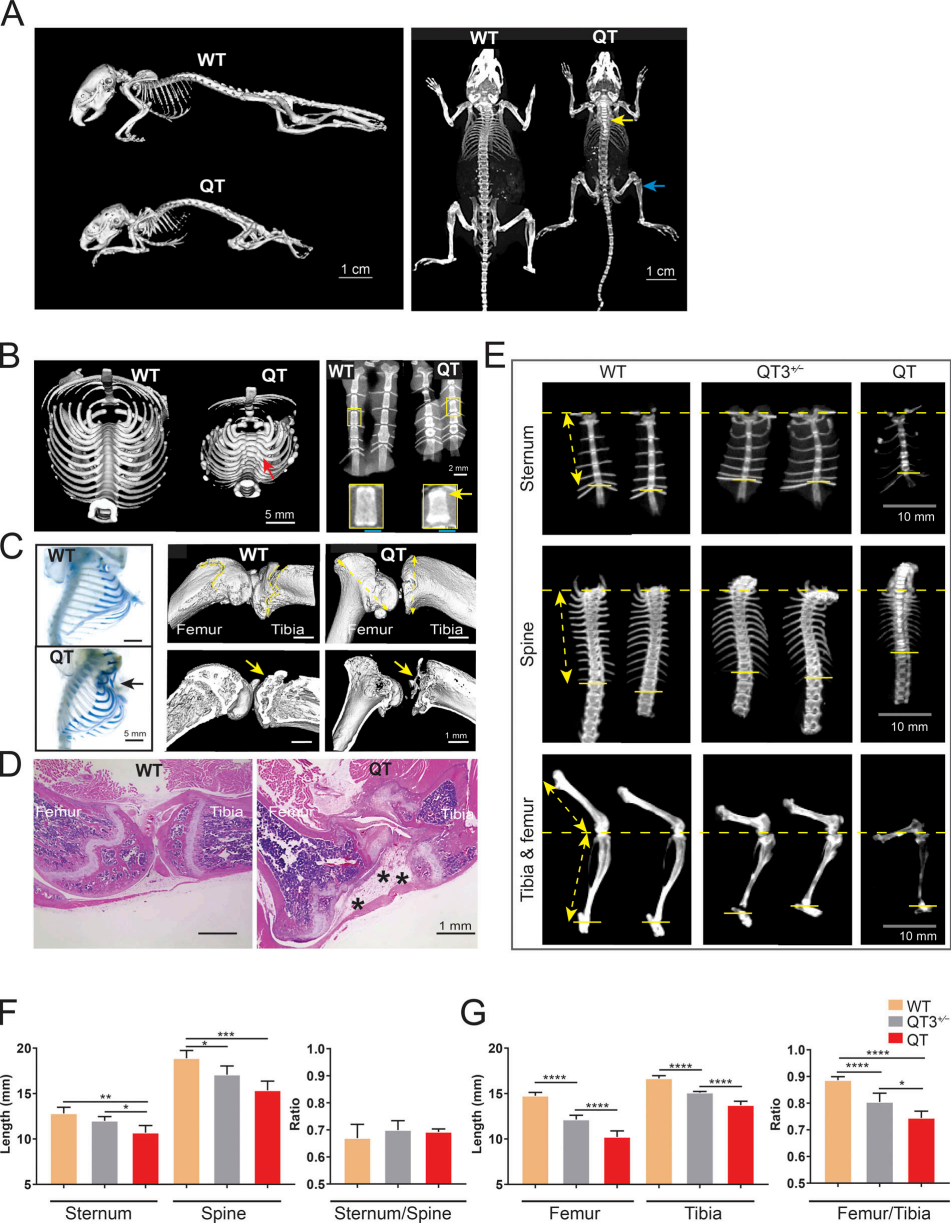
**TIMPs are required for postnatal growth and long bone isometry. (A)** Micro-CT images of WT and QT mice skeleton (age 8 wk) depict the short stature of QT mice as well as altered bone density (yellow arrow, brighter bone indicates higher density; blue arrow, lower density) in axial and appendicular bones. **(B)** Micro-CT scan of the thoracic girdle (left panel), presented after 3D isosurface rendering (4-wk-old WT and QT mice; transverse view). Red arrow points to a bony enlargement of rib-heads in QT mice. Faxitron x-ray images of 4-wk-old sternums (2 WT and 2 QT; right panel). The bright bands seen in enlargement reflect calcification at the edges of QT sternebrae (yellow arrow). **(C)** Alcian blue–stained thoracic girdles of 7-wk-old mice display Pectus formation in sternum of QT mice (black arrow; left panel). Micro-CT images of knee joints of 7-wk-old WT and QT mice (right panel). QT mice show metaphyseal flaring (double-sided yellow arrow) in femur (distal) and tibia (proximal) head. Yellow arrow indicates epiphysis of WT and loss of epiphysis in QT knee bone. **(D)** H&E-stained images of knee joints of 7-wk-old WT and QT mice display structural abnormality of QT knee joint. QT joint shows altered epiphysis (*), accompanied by fibrosis (4×/0.5-NA objective). **(E)** Micro-CT images of representative sternums (top), spines (middle), and leg bones (lower) of 8-wk-old WT, QT3^+/−^, and QT mice, depicting shortening of bone in TIMP-deficient mice. The distance from the top of sternum to the last attached rib was used to determine the length of sternum in different genotypes (double-sided yellow arrow). Similarly, the distance from the first attached rib to the last attached rib was measured to determine thoracic spine length for comparative purposes (double-sided yellow arrow). Leg bone length measurement depicted by double-headed yellow arrow. **(F)** Axial bone (sternum and thoracic spine) length and their proportionality in 8-wk-old WT (*n* = 7), QT3^+/−^ (*n* = 5), and QT (*n* = 3) mice. Mean values of each dataset are plotted in graphs with error bars representing SEM. Datasets were compared by one-way ANOVA following Sidak’s multiple comparison test, *, P < 0.05; **, P < 0.01; ***, P < 0.001; ****, P < 0.0001. **(G)** Leg bone (femur and tibia) length and their proportionality in 8-wk-old WT (*n* = 7), QT3^+/−^ (*n* = 5), and QT (*n* = 3) mice. Mean values of each dataset are plotted in graphs with error bars representing SEM. Datasets were compared by one-way ANOVA following Sidak’s multiple comparison test: *, P < 0.05; **, P < 0.01; ***, P < 0.001; ****, P < 0.0001.

Adult QT mice have short stature compared with WT or QT3^+/−^ littermates and exhibit a vivid skeletal phenotype (Fig. 2 A). Micro-CT imaging of the thoracic girdle exposed gross defects in bone architecture: a curved spine and bulging ribs at the costovertebral/costotransverse joints. Additionally, the sternum was shorter and displayed bright bands of calcification at the margins (Figs. 2 B and S1), with a small proportion (∼10%) of mice presenting pectus excavatum (Fig. 2 C), a sunken sternum, which is the most common congenital defect of the chest wall in humans (Tocchioni et al., 2013). Long bone defects were also prominent in adult QT mice, with their joints showing abnormal morphology and disappearance of the epiphysis such that only its remnants are visible by 7 wk of age (Fig. 2, C and D). Axial and appendicular bone segments, specifically the length of sternum, thoracic spine, femur, and tibia in WT, QT3^+/−^, and QT cohorts, were measured (Fig. 2 E). While both femur and tibia were smaller, a decrease in the femur/tibia ratio indicated a more extensively shortened femur in the QT mouse, whereas the ratio of sternum/thoracic spine were similar to controls (Fig. 2, F and G). These data demonstrate a role for the TIMP family in normal postnatal development of mammalian skeleton and isometric scaling of long bones.

### Growth plate closure in quadruple TIMP-deficient mice

**Figure 3.**
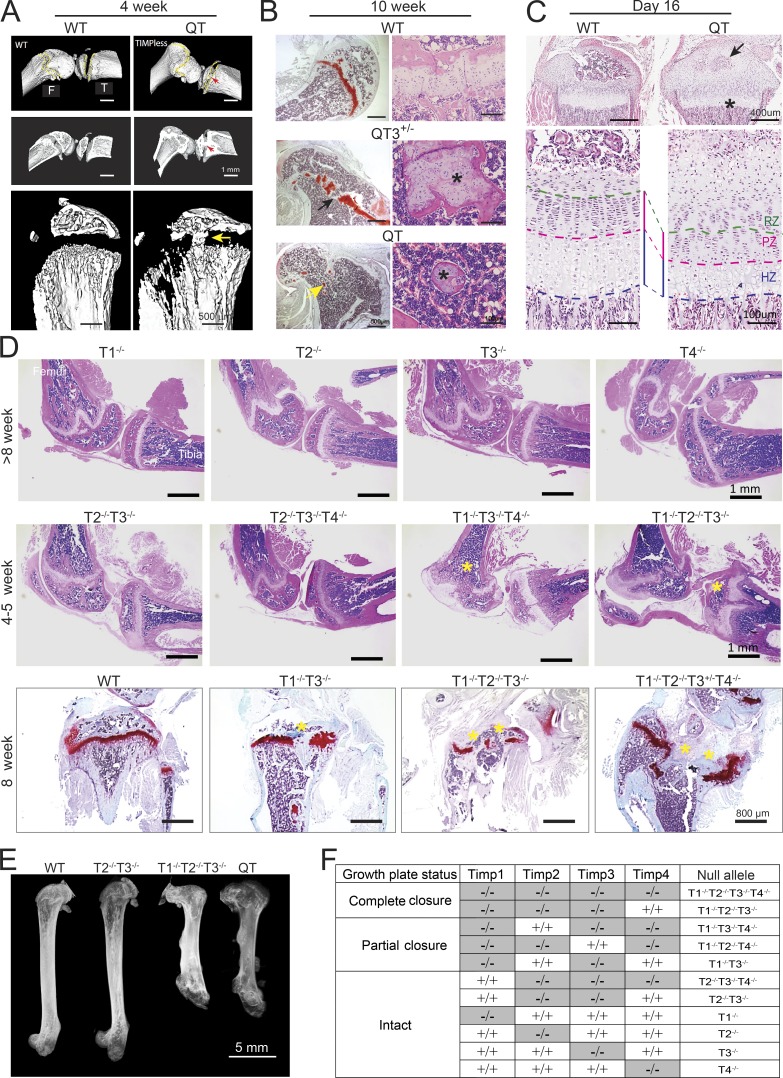
**Growth plate closure in different combination of TIMP knockouts. (A)** Micro-CT images of 4-wk-old WT and QT knee joint. QT mice exhibit growth plate closure in the tibia and femur (upper and middle panels; cross section view; red arrow). High-resolution micro-CT images demonstrating growth plate closure in QT tibiae (yellow arrow; lower panel). **(B)** Femurs stained with H&E (right panel) and safranin-O (left panel) from 10-wk-old mice. WT mice retain an intact growth plate; QT3^+/−^ cartilage is bridged by bone in several locations (black arrow), and only small islands of bone-encased cartilage remain in QT3^+/−^ and QT bone (yellow arrow), as shown at higher magnification (*) on right panel. **(C)** H&E-stained tibiae of 16-d WT and QT mice. Upper panels indicate impaired secondary ossification center (arrow) and flattened growth plate (*) in QT tibia. Enlarged view in lower panels indicates that QT tibia has thinner hypertrophic zone (HZ) and proliferating zone (PZ). RZ, resting zone. **(D)** H&E-stained knee joints of different TIMP knockout combinations. Upper panels display knee joints of individual TIMP knockouts. Middle panels depict different levels of growth plate closure (*) of multiple-TIMP knockouts. Safranin O–stained tibiae of 8-wk-old WT and multiple TIMP knockouts (lower panels) exhibit growth plate closure (*; 4×/0.5-NA objective). **(E)** Faxitron x-ray image of femur, demonstrating the lengths of WT and different multiple TIMP knockouts (8-wk-old). **(F)** Summary of TIMP knockout combinations that result in growth plate closure or have an intact growth plate.

**Figure 4.**
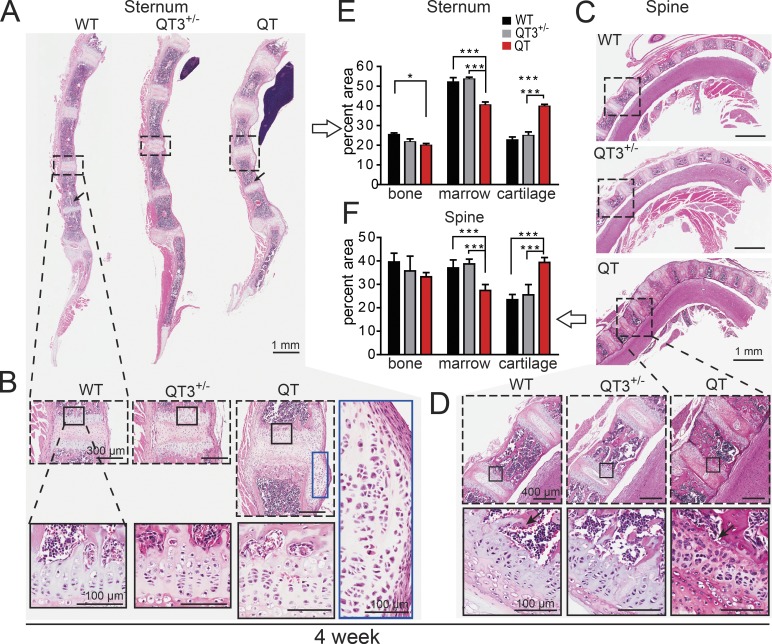
**TIMPs are required for normal morphology of axial bone. (A–D)** H&E-stained sagittal plane section of sternum (A and B) and spine (C and D; cervical and thoracic vertebrae) from 4-wk old mice. **(B and D)** Marked areas compare a single cartilaginous joint bordered by two growth plates. Further magnification exhibits cellular organization in growth plate cartilage and laterally migrated cartilage of QT sternum. Black arrow indicates loss of trabecular bone. **(E and F)** Histomorphometric quantification of percentage of cartilage, bone, and marrow in sternums and spine (7–8 wk-old; *n* = 3–6/group). ANOVA with Bonferroni’s multiple comparison test assessed significance for each tissue. *, P < 0.05; ***, P < 0.001.

The epiphyseal growth plate is the developmental region responsible for bone elongation. The growth plate undergoes progressive narrowing and closure concomitant with pubertal growth in humans, although it does not fuse in mice (Börjesson et al., 2012; Staines et al., 2018). We examined the appendicular and axial skeleton (Fig. 3 and 4). We observed cartilage overabundance as well as bone bridges across the ossification centers of long bones, resulting in aberrant growth plate closure in quadruple TIMP-deficient mice (Fig. 3 A). Chondrocyte columnar organization in this region was completely disrupted as early as 4 wk, with only small bone-encased islands of cartilage remaining at 10 wk (Fig. 3 B), like those seen in a fusing human pubertal growth plate. QT3^+/−^ mice showed a less severe phenotype (Fig. 3 B), although the growth plates of axial bones, sternum, and spine were also abnormal. Histology revealed an excess of sternal cartilage, which extended beyond the bone margins in 4-wk-old QT mice (Fig. 4, A and B). Similar changes occurred in the cervical and thoracic vertebrae (Fig. 4, C and D). Histomorphometry confirmed cartilage hyperplasia (1.7-fold) at the expense of highly compacted bone marrow cavities in both sternum and spine (Fig. 4, E and F). Overall, the absence of TIMP disrupted the postnatal chondrocyte program in both the axial and appendicular skeleton.

We then tracked skeletal development from the late embryonic to the prepubertal stage. TIMP-deficient groups showed a small reduction in rib cage and tibial length at embryonic day 17.5 (E17.5; Fig. S2, A–C), although long bone growth plates were indistinguishable between these groups at postnatal days 2 and 7 (Fig. S3, A and B). By postnatal day 16, we observed a marked reduction in proliferative and hypertrophic chondrocyte zones (Fig. 3 C) and delayed development of secondary ossification centers in QT, pointing to a defective chondrocyte maturation program. Chronological analysis of long bones and their growth plates demonstrates that the major deformities in TIMP-deficient bones arise postnatally, although the possibility of a subtle embryonic phenotype remains. A similar change in growth plate has previously been reported in mice that harbor an FGFR3 activating mutation, with concomitant lowering of *Ihh* expression (Naski et al., 1998; Wang et al., 1999).

### TIMP1 is critical for sustaining normal growth plate

We investigated which of the four TIMPs are important for normal growth plate structure by evaluating the length of long bones and growth plate closure phenotypes of individual TIMP knockouts as well as several combinatorial knockouts (Fig. 3, D–F). A single TIMP deficiency had no impact on the growth plate (Fig. 3 D) or long bone length (Fig. S3 C). Bone bridges were readily seen in each combination lacking TIMP1 (i.e., T1^−/−^ T3^−/−^, T1^−/−^ T2^−/−^ T3^−/−^, and T1^−/−^ T3^−/−^ T4^−/−^; Fig. 3 D), whereas compound T2^−/−^ T3^−/−^ T4^−/−^ mice had an intact growth plate (Fig. 3 D). Interestingly, the only two T2^−/−^ T3^−/−^ mice that serendipitously survived in utero lethality exhibited only a minor distortion in the growth plate without bone bridge development (Fig. 3 D) and no alteration in bone length (Fig. 3 E). Partial or complete growth plate aberration in specific genotypes is summarized in Fig. 3 F. These data collectively show that TIMP1 along with TIMP3 is crucial for growth plate integrity.

### Differential rescue of long bone proportionality by MMP- and ADAMTS-resistant aggrecans

**Figure 5.**
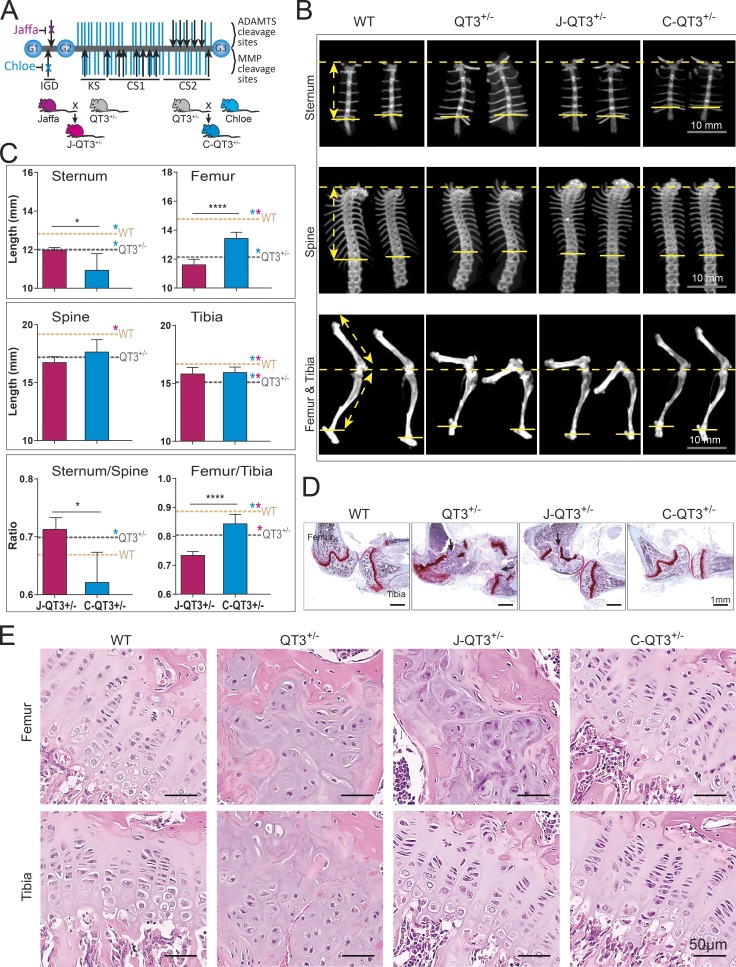
**Metalloprotease-resistant aggrecan rescues long bone proportionality. (A)** Structure of aggrecan and the Chloe/Jaffa knock-in mutation. The aggrecan protein core (gray) contains three globular regions (G1–G3) and has ∼100 glycosaminoglycan chains (purple) attached. MMP and ADAMTS cleavage sites are indicated by black arrows. Disruption of specific cleavage sites by knock-in mutations are indicated by an x. Schematic of breeding strategies to generate J-QT3^+/−^ and C-QT3^+/−^ mice. CS1, chondroitin sulfate domain 1; CS2, chondroitin sulfate domain 2; KS, keratan sulfate. **(B)** Representative micro-CT images used to measure the length of sternum, the spine, and the leg bones of 8-wk old WT, QT3^+/−^, C-QT3^+/−^, and J-QT3^+/−^. Sternum (upper panel), spine (middle panel), and leg bones (lower panel). Arrows indicate measurements as described in legend of Fig. 1 H. **(C)** Quantification of axial and appendicular bones length in 8-wk-old WT, QT3^+/−^, C-QT3^+/−^, and J-QT3^+/−^. The sternum, spine, femur, and tibia are all shorter in the QT3^+/−^ mouse compared with WT controls (dashed lines: WT, orange; QT3^+/−^, gray). C-QT3^+/−^ mice have shorter sternums. Sternum:thoracic spine ratio indicates the effect was more pronounced in sternum than spine. C-QT3^+/−^ partially rescued the shortened QT3^+/−^ femur length, but J-QT3^+/−^ mice could not. The femur:tibia ratio reflects that C-QT3^+/−^ mice partially restored the leg bone compared with QT3^+/−^ (*n* = 5–7/each group). Mean values of each dataset are plotted in graphs with error bars representing SEM. Datasets were compared by unpaired *t* test, *, P < 0.05; ****, P < 0.0001. Dunnett’s test P < 0.05 when WT or QT3^+/−^ compared with C-QT3^+/−^ (blue*) or J-QT3^+/−^ (pink*). **(D)** Safranin O–stained knee joints of WT, QT3^+/−^, C-QT3^+/−^, and J-QT3^+/−^ mice at 8 wk of age. Black arrows show growth plate closure in femur and tibia of QT3^+/−^ mice; femur growth plate is rescued in the C-QT3^+/−^ but not in J-QT3^+/−^, while the tibia growth plate is rescued by both mutant aggrecan (4×/0.5-NA objective). **(E)** H&E-stained femur and tibia growth plates of WT, QT3^+/−^, C-QT3^+/−^, and J-QT3^+/−^ mice (8-wk-old) chondrocyte disorganization in the growth plates of QT3^+/−^ femur and tibia, J-QT3^+/−^ femur but not in the C-QT3^+/−^ mice. Scale bar = 50 µm; 20×/0.5-NA objective.

Aggrecan is a core proteoglycan of the cartilage matrix and is critical for its load-bearing function. Mutations in aggrecan result in severely disrupted growth plates and skeletal deformities in humans, chickens, and mice (Kimata et al., 1981; Li et al., 1993; Warman et al., 2011). Aggrecan loss in articular cartilage is predominantly a proteolytic process mediated by MMP and ADAMTS proteases and has been extensively studied in arthritis (Roughley and Mort, 2014). Multiple metalloprotease cleavage sites are located in the interglobular domain (IGD) and the chondroitin sulfate–rich region of aggrecan (Fig. 5 A); proteolysis in the IGD releases the entire glycosaminoglycan-containing portion, with concomitant loss of mechanical properties, whereas proteolysis in the chondroitin sulfate rich region is part of normal aging and does not appear to affect biomechanics (Ilic et al., 1998). We used a genetic approach to test the contribution of aggrecan cleavage to the skeletal abnormalities seen in TIMP-deficient mice by crossing in Chloe or Jaffa knock-in mutations that block either MMP (Chloe) or ADAMTS (Jaffa) cleavage sites in the IGD of aggrecan (Little et al., 2005, 2007), as modeled in Fig. 5 A. Specifically, amino acid sequence mutation ^342^FFG to ^342^GRT eliminates the MMP-cleavage site at N^341^/F^342^ in Chloe; and ^374^ALGS to ^374^NVYS eliminates the ADAMTS cleavage site at E^373^/A^374^ in Jaffa. Chloe and Jaffa mice were crossed with QT3^+/−^ mice to produce C-QT3^+/−^ and J-QT3^+/−^ cohorts.

We found that incorporation of the Chloe mutation rescued the shortening of both the tibia and femur of QT3^+/−^ mice, demonstrating that TIMP regulation of MMP activity at this site is necessary for normal postnatal growth plate development. Surprisingly, the Jaffa mutation rescued the tibia but not the femur length (Fig. 5, B and C), showing a differential requirement for regulation of ADAMTS activity in these two long bones. Consistent with the report that the distal femur and proximal tibia growth plates are the main contributors to bone elongation (Serrat et al., 2007), C-QT3^+/−^ growth plates displayed normal histology at both these sites, while the J-QT3^+/−^ femur growth plate still had a bone bridge, in keeping with its shortened length (Fig. 5 D). This site of growth plate closure was accompanied by disorganized chondrocytes in the J-QT3^+/−^ femur, although normal chondrocyte columnar architecture was seen in the three rescued growth plates: C-QT3^+/−^ femur, C-QT3^+/−^ tibia, and J-QT3^+/−^ tibia (Fig. 5 E). Furthermore, metaphyseal flaring of the proximal tibia head and the distal femoral head were rescued in C-QT3^+/−^, but not in the J-QT3^+/−^ femur (Fig. 5 D). Finally, we determined the femur:tibia ratios as a measure of bone proportionality across WT, QT3^+/−^, C-QT3^+/−^, and J-QT3^+/−^ cohorts (Fig. 5, B and C), which revealed that individual growth plates of long bones are differentially dependent on protease activity. This comparison points to the importance of TIMPs’ control over both MMP and ADAMTS during growth of the appendicular skeleton.

### Exacerbation of sternum defect by MMP-resistant aggrecan

We also evaluated the outcome of blocking MMP- and ADAMTS-mediated aggrecan cleavage in axial bones, in the context of TIMP loss. The length of the thoracic vertebral column and sternum were measured in all cohorts (Fig. 5, B and C). Axial bone length did not further change with the incorporation of ADAMTS-resistant aggrecan in the J-QT3^+/−^ cohort, whereas MMP-resistant aggrecan (C-QT3^+/−^) had the unexpected and dramatic effect of further shortening the sternum. The exceptionally short sternum observed in C-QT3^+/−^ mice, compared with J-QT3^+/−^ and QT3^+/−^ mice, demonstrates the requirement for MMP-mediated cleavage of aggrecan for normal sternum growth. Histologically, C-QT3^+/−^ mice also displayed thick fibrocartilaginous pads between the sternebrae (Fig. S4). The uneven rescue of axial bone segment shortening with MMP-resistant aggrecan is highlighted by the sternum:spine ratio (Fig. 5 C). These data demonstrate that postnatal growth of skeletal segments requires natural metalloprotease inhibitor regulation of MMP and ADAMTS processing of aggrecan.

### Expression profiling reveals IHH and FGF-2 deregulation in chondrocytes lacking TIMPs

**Figure 6.**
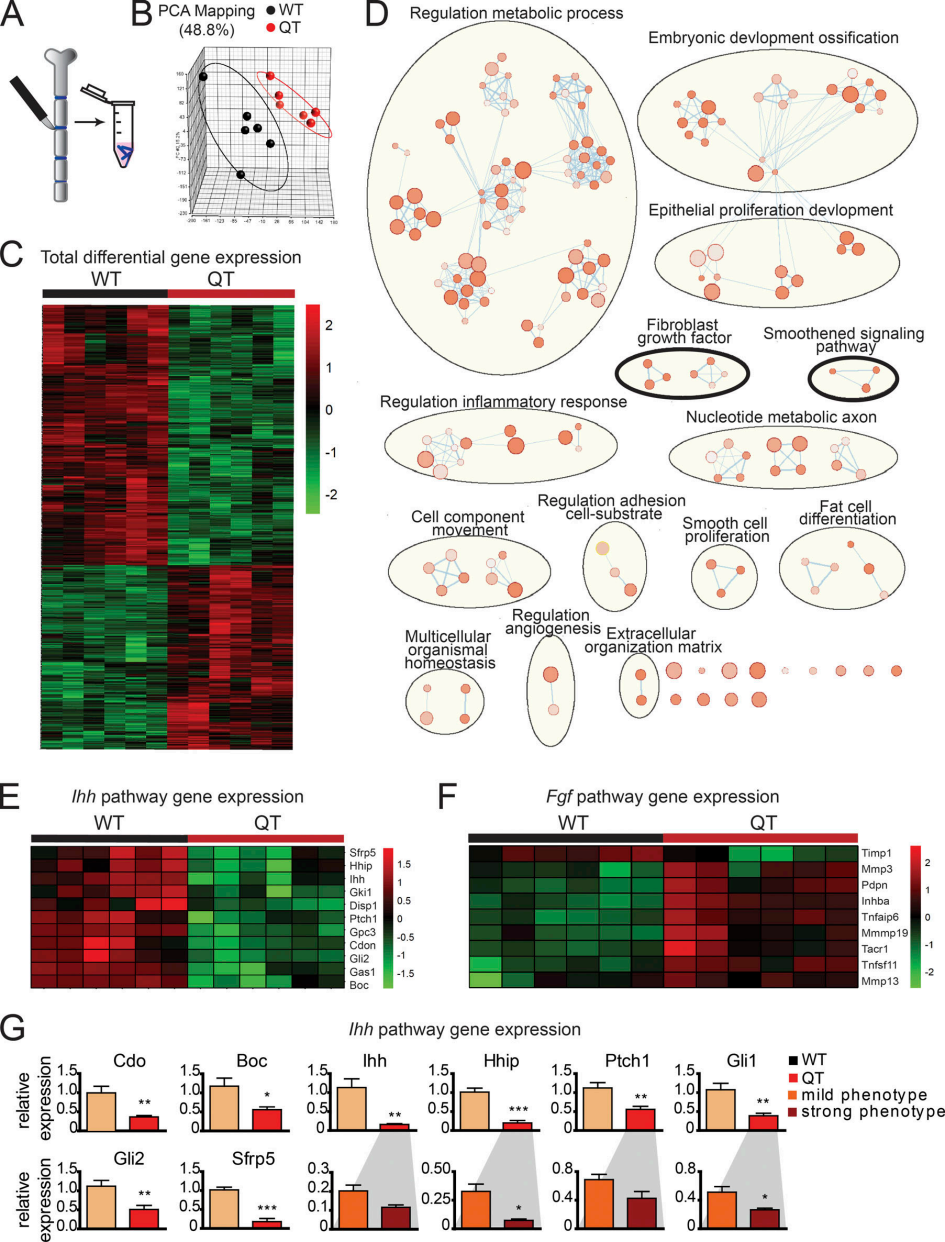
***Ihh* down-regulation and higher FGF-2 signaling in QT chondrocytes. (A)** Schematic of sternal cartilage macrodissection and RNA isolation. **(B)** Principal component analysis of microarray data from cartilage displaying distinct groups by genotype. Each datapoint represents one mouse. **(C)** Heat map of normalized mRNA levels of the 625 significantly altered genes between WT and QT samples (*n* = 6 each) determined by linear modeling with an adjusted P-value threshold of 0.05 and absolute fold change ≥1.5. **(D)** Gene enrichment analysis depicts altered biological processes in QT cartilage compared with WT. This analysis indicates a change in the bone development program in QT mice involving the IHH (smoothened) and FGF (encircled with black thick line) pathways. Size of nodes indicates number of genes involved, and color intensity indicates the q value (darker color indicates lower q value; q value cutoff is <0.05). **(E)** Heatmap of normalized mRNA levels of the IHH signaling pathway components, which are altered in QT versus WT samples. **(F)** Heatmap of normalized mRNA levels of FGF-2 target genes, in WT and QT cartilage. **(G)** Quantitative PCR confirming IHH pathway down-regulation (*n* = 6/genotype). Expression of genes *cdo, Boc, Ihh, Hhip, Ptch1, Gli1, Gli2*, and *Sfrp5.* Expression of *Ihh, Hhip*, *Ptch1,* and *Gli1* were further subdivided by the severity of QT sternal phenotype (*n* = 3/group). Mean values of each dataset are plotted in graphs with error bars representing SEM. Data were compared using unpaired *t* test: *, P < 0.05; **, P < 0.01.

To seek the mechanism underlying the disrupted chondrocyte maturation program, we macrodissected sternal cartilage excluding the xiphoid process for expression profiling (Fig. 6 A); *Timp* genotypes were confirmed before transcriptome analyses. Principal component analysis segregated WT and QT groups, underscoring broad differences in their gene expression profiles (Fig. 6 B); a total of 625 genes were differentially expressed in their sternal cartilage (P ≤ 0.05, fold change ≥1.5; 362 down-regulated and 263 up-regulated; Fig. 6 C and Table S1). Functional enrichment analysis using g:Profiler pointed to the smoothened and FGF pathways along with alterations in metabolism, embryonic development, inflammatory response program, and others (Fig. 6 D). Additionally, KEGG (Kyoto Encyclopedia of Genes and Genomes) pathway enrichment using Enrichr also pointed to IHH as the top down-regulated pathway in TIMP-deficient cartilage (Fig. S5 A). IHH is a major regulator of chondrocyte maturation (St-Jacques et al., 1999). It binds to the receptor Ptch1, relieving inhibition of Smo to initiate signaling, an interaction promoted by the coreceptors Cdo, Boc, and Gas1 and hindered by Gpc3 and Hhip (Briscoe and Thérond, 2013). Quantitative PCR verified the down-regulation of these IHH pathway components in QT cartilage (Fig. 6, E and G), whereby the extent of decrease in the canonical IHH target genes, *Gli1, Hhip*, and *Ptch1*, correlated with phenotype severity. The enrichment map also pointed to FGF-2 signaling up-regulation in QT samples (Fig. 6 D). Chondrocytes express multiple genes in response to FGF-2 stimulation such as *Inhba*, *Mmp3*, *Mmp19*, *Pdpn*, *Tnfaip6, Tnfsf11*, and *Timp1* (Chong et al., 2013); several of these genes were increased in TIMP-deficient sternal cartilage (Fig. 6 F).

**Figure 7.**
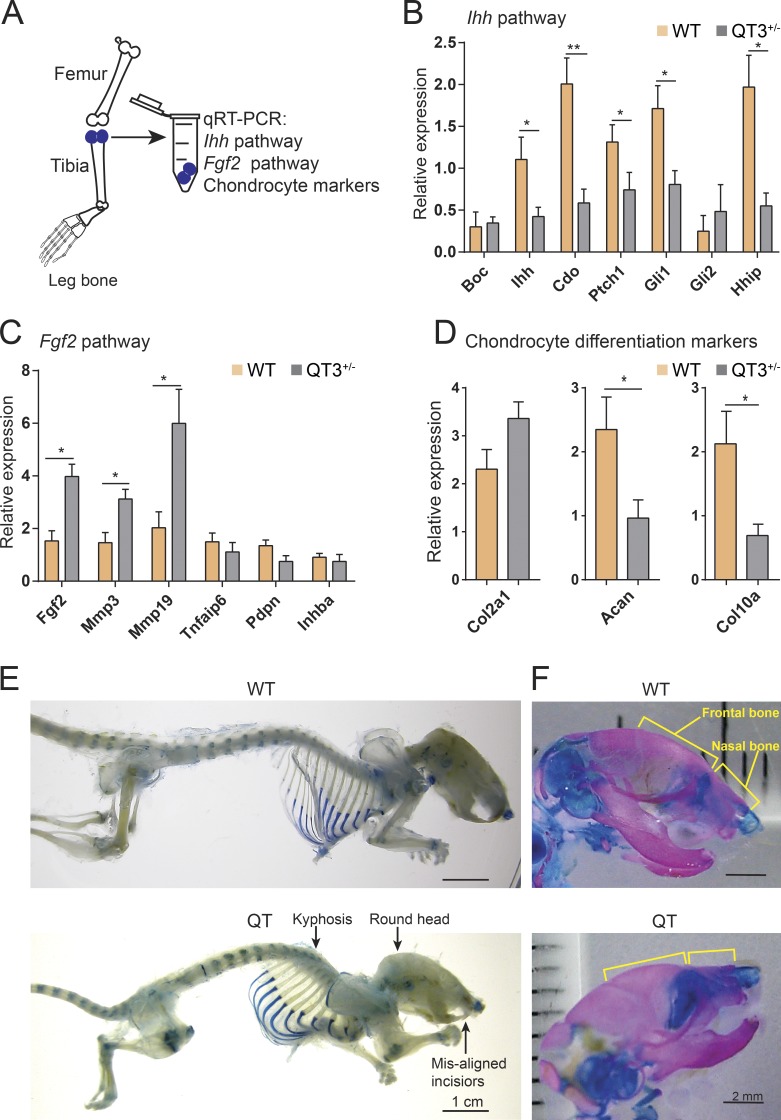
***Ihh* down-regulation and higher FGF-2 signaling in long bone chondrocyte. (A)** Schematic of proximal tibial head RNA isolation for gene expression analysis. **(B)** Analysis of IHH pathway genes (*Boc, Ihh, Cdo, Ptch1, Gli1, Gli2, Hhip*) RT-PCR expression in proximal head of tibia of 4-wk-old WT and QT3^+/−^ mice (*n* = 5/group). Mean values of each dataset are plotted in graphs with error bars representing SEM. Data were compared using unpaired *t* test: *, P < 0.05; **, P < 0.01. **(C)** Expression (RT-PCR) of FGF-2 pathway genes (*Fgf2*, *Mmp3*, *Mmp9*, *Tnfaip6*, *Pdpn*, and *Inhba*) in proximal head of tibia of 4-wk-old WT and QT3^+/−^ mice (*n* = 4 for WT; *n* = 5 for QT3^+/−^). Mean values of each dataset are plotted in graphs with error bars representing SEM. Data were compared using unpaired *t* test: *, P < 0.05. **(D)** Chondrocyte development marker gene (*Col2a1*, *Acan*, and *Col10a1*) expression in proximal head of tibia of 4-wk-old WT and QT3^+/−^ mice (*n* = 5/group). Values represent mean ± SEM; *, P < 0.05; **, P < 0.01. Mean values of each dataset are plotted in graphs with error bars representing SEM. Data were compared using unpaired *t* test: *, P < 0.05. **(E)** Alcian blue-stained skeleton of 8–9-wk-old WT and QT mice depicting round head, kyphosis, and misaligned incisors in QT mice. Scale bar = 1 cm. **(F)** Alcian blue– and alizarin red–stained skull of 1-d-old WT and QT mice depicting shortening of nasal and frontal bone. Scale bar = 2 mm.

We then assessed the proximal tibia head to determine whether TIMP-deficient long bones had mechanistic alterations similar to the sternal cartilage. *Ihh* down-regulation and *Fgf2* up-regulation was confirmed in the growth plate cartilage containing region of the QT3^+/−^ long bones (Fig. 7, A–D). Specifically, *Mmp3* and *Mmp19* were up-regulated and *Ihh* and *Acan* were down-regulated as the FGF-2 target genes. Furthermore, several markers of chondrocyte maturation (resting, collagen II; proliferating, aggrecan; hypertrophic, IHH and collagen X) were found to be altered, consistent with the reduction of hypertrophic chondrocyte zone in TIMP-deficient long bones (Figs. 3 C and 7 D). Increased FGF-2 signaling prompted us to look for additional skeletal changes in the quadruple TIMP-deficient mice. We noted shortening of the nasal and frontal bones, kyphosis, and misaligned closure of the upper and lower incisors (Fig. 7, E and F), phenotypes similar to those reported in mice harboring an FGFR3 activating mutation (Wang et al., 1999). Collectively, TIMPless chondrocytes have both decreased IHH and increased FGF-2 growth factor signaling in both sternum and long bone growth plates.

### TIMPs regulate FGF-2–dependent *Ihh* expression in chondrocytes

**Figure 8.**
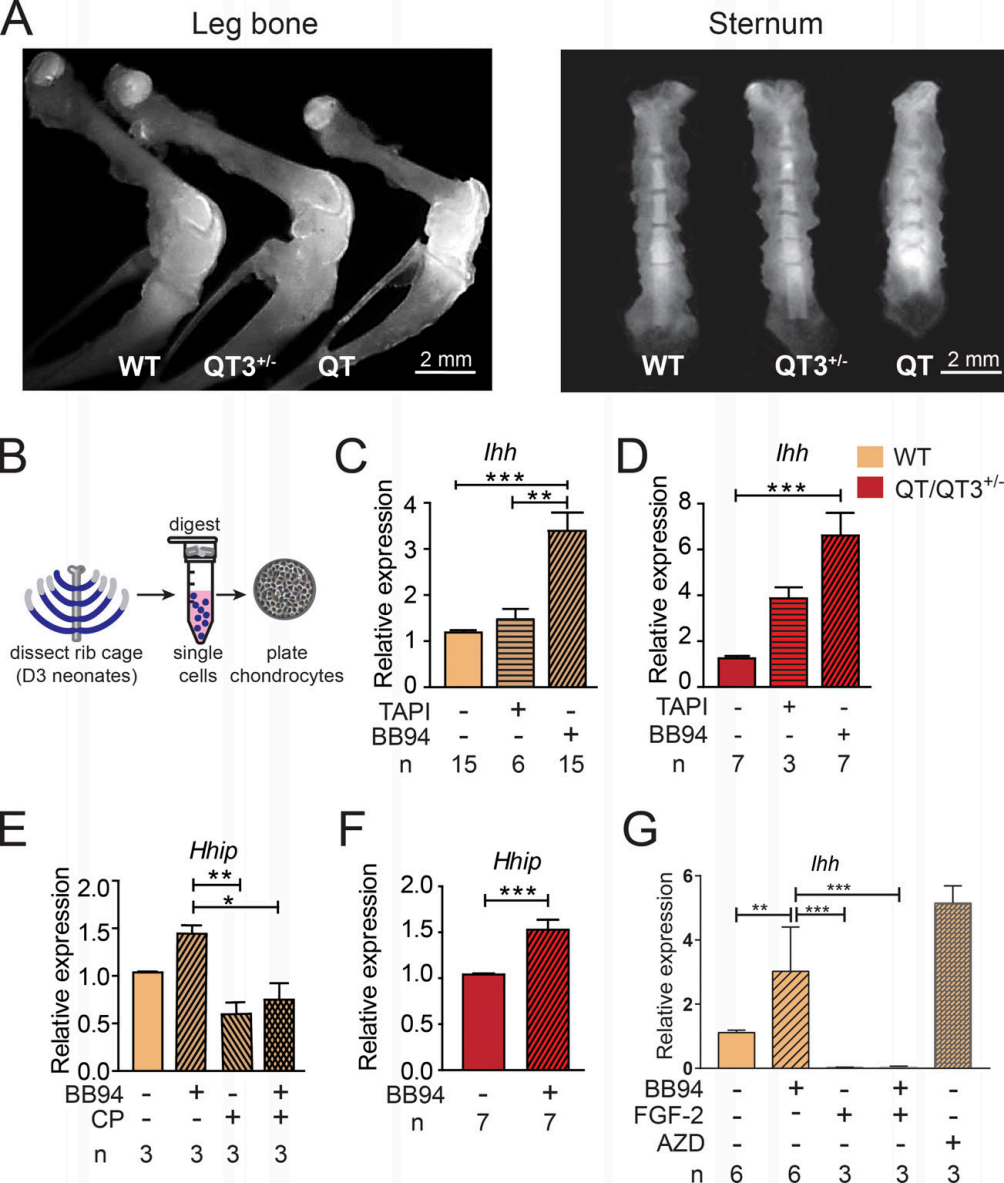
**TIMP/FGF-2/IHH comprise a critical axis for the growth plate integrity. (A)** Metalloprotease activity detected in 4-wk-old bone, following in vivo injection of MMPSense750 fluorescent beacon. Ex vivo, growth plates of leg bone (left panel) display enhanced metalloprotease activity with progressively higher signal in QT3^+/−^ and QT than WT. QT3^+/−^ and QT sternum (right panel) also have higher metalloprotease activity than WT. **(B)** Schematic of chondrocyte isolation and culture. **(C–F)** Expression of *Ihh* and *Hhip* in chondrocyte cultures determined by RT-PCR. **(C and D)** *Ihh* expression following DMSO, BB94 (10 µM), or TAPI (20 µM) treatment in WT (C) and QT/QT3^+/−^ (D) cultured chondrocytes. **(E and F)** *Hhip* expression following DMSO, BB94, or cyclopamine (CP; 10 µM) treatment in WT (E) and QT/QT3^+/−^ (F) cultured chondrocytes. All reagents with media changed every 48 h over 7 d (n indicates number of independent mice). Mean values of each dataset are plotted in graphs with error bars representing SEM. Data were compared using one-way ANOVA following Dunnett’s multiple comparison test (C and D), Bonferroni’s multiple comparison test (E), and unpaired *t* test (F): **, P < 0.01; ***, P < 0.001. **(G)** *Ihh* expression following DMSO, BB94 (10 µM), FGF-2 (10 ng/ml), FGF-2 + BB94 (10 ng/ml + 10 µM), or AZD4547 (FGFR inhibitor; 500 nM) treatment in WT chondrocyte culture. All reagents with media changed every 48 h over 7 d (n indicates number of independent mice). Mean values of each dataset are plotted in graphs with error bars representing SEM. Datasets were compared by one-way ANOVA following Sidak’s multiple comparison test: *, P < 0.05; **, P < 0.01; ***, P < 0.001.

Next, we used two probes designed to detect metalloprotease activity in vivo. Radioactive, pan-MMP phosphinic pseudopeptide (^3^H-217; high-affinity binding to the active site of most MMPs) showed a heightened signal in the skeleton of QT mice (Fig. S5 B). Likewise, ex vivo imaging of organs from mice injected with MMPSense750 (fluorescent pan-MMP beacon upon cleavage) highlighted skeleton, with an intense signal in the growth plate. The long bones, sternum, and spine of TIMP-deficient mice displayed elevated MMP activity (Figs. 8 A and S5 C).

To address the causal relationship between increased metalloprotease activity and down-regulation of the IHH pathway in TIMP-deficient cartilage, we set up primary chondrocyte cultures derived from sternum and ribs of neonatal pups (Fig. 8 B). Alcian blue staining indicated the production of matrix component by chondrocytes in vitro, attesting to their functional capacity (Fig. S5 D), and we verified that TIMPs are expressed in WT chondrocytes (Fig. S5 E). These cultures were treated with two metalloprotease inhibitors, either a broad-spectrum inhibitor (BB94) or one with specificity for adamalysins (TAPI-1; Fig. 8, C–F). We found that WT chondrocytes responded to BB94 by significantly elevating *Ihh* expression (Fig. 8 C) and by partially up-regulating *Hhip* expression (Fig. 8 E), whereas the IHH pathway inhibitor cyclopamine (a control) blocked the induction of *Hhip* (Fig. 8 E). TIMP*-*deficient chondrocytes responded to both metalloprotease inhibitors with a far greater induction of *Ihh* and *Hhip* expression (Fig. 8, D and F). These data indicate that metalloproteases normally act to negatively regulate IHH signaling in chondrocytes, and blocking the excess metalloprotease activity in TIMP-deficient chondrocyte relieves the *Ihh* suppression.

FGF-2 signaling in chondrocytes is known to suppresses *Ihh* expression, and both are implicated in various bone pathologies (Su et al., 2014). For instance, transgenic FGF-2 overexpression results in dwarfism in mice (Coffin et al., 1995), and activating FGFR3 mutations lead to achondroplasia and shortened long bones with disorganized chondrocyte columns in growth plate (Chen et al., 1999; Lee et al., 2017). We therefore probed the effect of metalloprotease inhibitors on *Ihh* expression in the presence or absence of recombinant FGF-2 in chondrocyte cultures (Fig. 8 G). FGF-2 treatment lowered *Ihh* expression whereas pan-FGFR inhibitor AZD4547 induced *Ihh* indicating the presence of endogenous FGF-2 signaling in this culture system. Importantly, combining BB94 with FGF-2 did not further alter *Ihh* expression suggesting that metalloprotease activity does not influence the bioactivity of exogenously added FGF-2.

### Metalloprotease-resistant aggrecans alter FGF-2 localization and release

**Figure 9.**
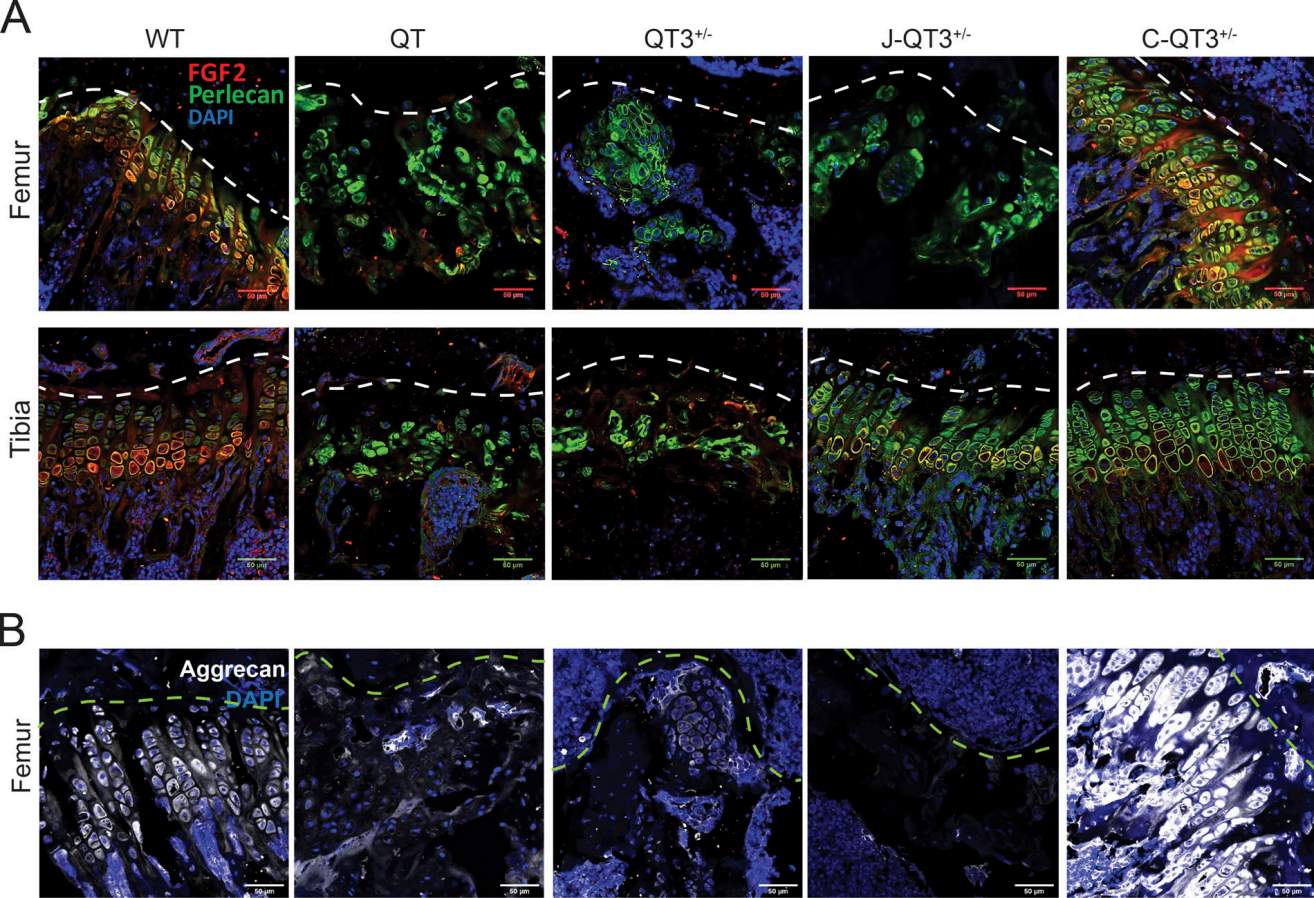
**Protease-resistant aggrecans and FGF-2 levels. (A)** Immunostaining of FGF-2 and perlecan in the femur (top panel) and tibia (bottom panel) of 8-wk-old WT, QT, QT3^+/−^, J-QT3^+/−^, and C-QT3^+/−^ mice. FGF-2 staining and perlecan organization are restored in growth plate of C-QT3^+/−^ femur but not of J-QT3^+/−^, and in the tibia growth plate in both J-QT3^+/−^ and C-QT3^+/−^ mice. Scale bar = 50 µm; 20×/0.8-NA objective. **(B)** Immunofluorescence of aggrecan in the femur of 8-wk-old WT, QT, QT3^+/−^, J-QT3^+/−^, and C-QT3^+/−^ mice. Scale bar = 50 µm; 20×/0.8-NA objective.

**Figure 10.**
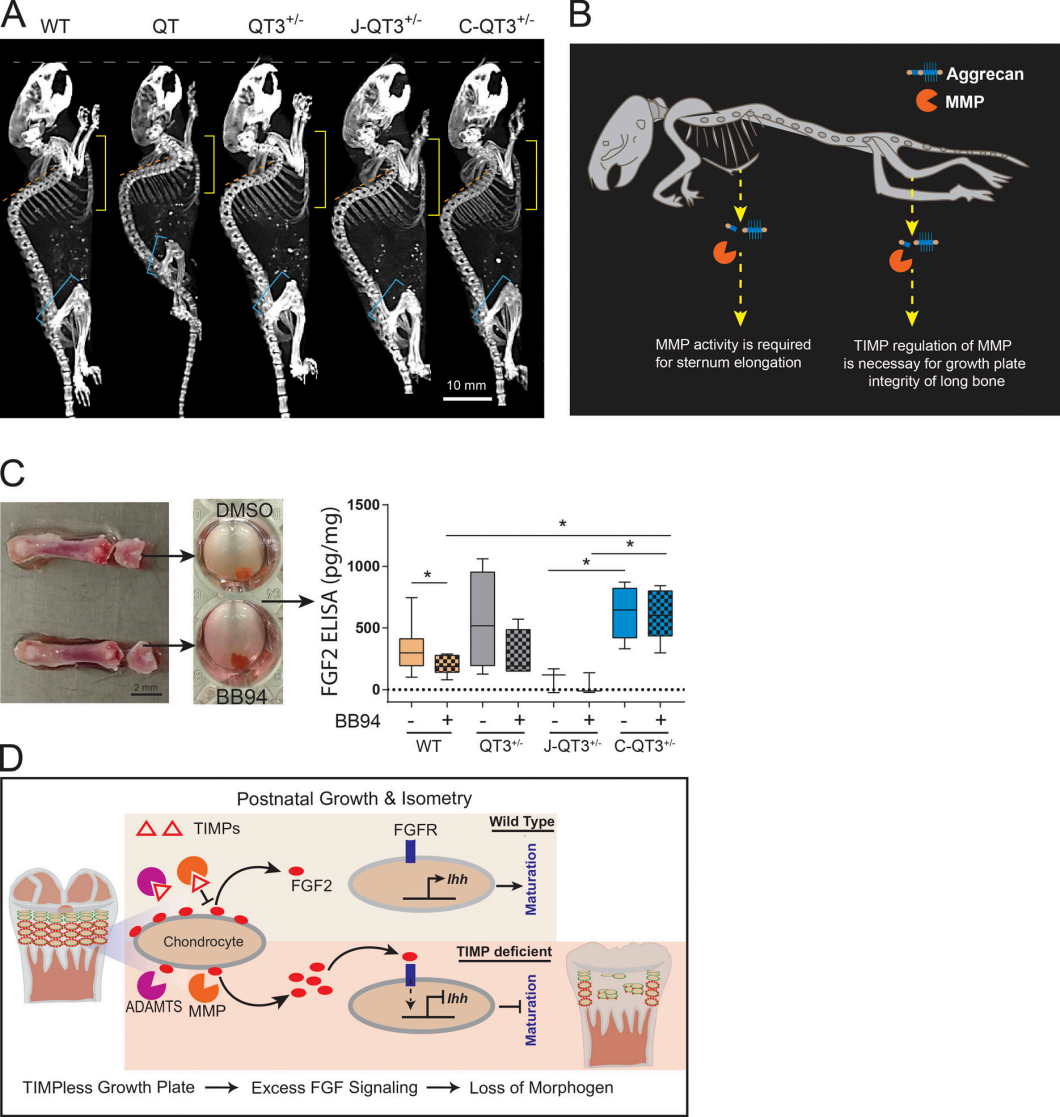
**Protease-mediated FGF-2 release and body stature. (A)** Whole-body micro-CT images of 8-wk-old WT, QT, QT3^+/−^, J-QT3^+/−^, and C-QT3^+/−^ mice. Images allow comparison of disproportion in the femur and tibia lengths, sternum, and spine curvature among different genotypes (yellow line, length of sternum; orange line, extent of kyphosis; blue line, femur length). Scale bar = 10 mm. **(B)** Cartoon depicting the effect of aggrecan cleavage by MMPs on bone length. The lack of MMP regulation by TIMPs (excess MMP activity) results in growth plate aberrations in the long bones, while the lack of MMP processing has the profound effect of shortening the sternum. **(C)** Distal femoral head explant culture to determine FGF-2 level by ELISA on the femoral distal head culture supernatant of WT (*n* = 10), QT3^+/−^ (*n* = 4), J-QT3^+/−^ (*n* = 3), and C-QT3^+/−^ (*n* = 5) mice. Reduction of FGF-2 release from contralateral femoral head by BB94 treatment, determined by ratio paired *t* test. Each box of box-and-whisker plot shows second to third quartile of datasets and line within the box shows median; *, P < 0.05. **(D)** Pictorial summary delineating the role of TIMPs in molecular processing to alter the bone length. Activation of the FGFR by excessive metalloprotease release of perlecan-bound FGF2 represses IHH-mediated chondrocyte maturation.

Perlecan is an FGF-2–binding heparan sulfate proteoglycan that localizes to the cell surface, and multiple MMPs are capable of releasing sequestered FGF-2 by cleaving perlecan (Whitelock et al., 1996; Tholozan et al., 2007). We reasoned that elevated FGF-2 bioavailability due to excess metalloprotease activity may be responsible for growth plate closure. FGF-2 and perlecan immunofluorescence staining to mark chondrocyte pericellular space in situ revealed colocalization of perlecan and FGF-2 on the chondrocyte cell surface in the growth plate of WT femoral and tibial distal heads (Fig. 9 A). Both chondrocyte organization and perlecan/FGF-2 colocalization were lost in the QT and QT3^+/−^ animals. The Jaffa and Chloe mutations restored chondrocyte organization and perlecan/FGF-2 colocalization in the tibia (Jaffa and Chloe) and femur (only Chloe; Fig. 9 A). Surprisingly, the Chloe mutation resulted in higher chondrocyte number and higher Perlecan/FGF-2 costaining, changes that were also reflected in the aggrecan staining pattern (Fig. 9 B). The differential rescue of chondrocyte organization by the Chloe and Jaffa mutations corresponded to the recovery in bone lengths in those mice (Fig. 5 C and Fig. 10, A and B).

The release of perlecan-sequestered FGF-2 from the pericellular space of chondrocytes is thought to be important for its bioactivity (Jonca et al., 1997; Gavrilovic, 2009). To determine whether FGF-2 release is affected in the TIMP-deficient or Chloe/Jaffa crosses, we set up the femoral distal head explant culture system (Stanton et al., 2011) and used ELISA to measure FGF-2 in the supernatants. The concentration of FGF-2 in the WT supernatant was lower in the presence of the metalloprotease inhibitor BB94 (Fig. 10 C). Although variable, the level of FGF-2 was higher in the supernatant of QT3^+/−^ than WT femur head explant cultures, consistent with the higher metalloprotease activity playing a role in FGF-2 release from the cell surface. J-QT3^+/−^ mice released far less FGF-2, consistent with there being less FGF-2 by immunofluorescence staining (Fig. 9 A), whereas Chloe mice released more FGF-2 into the media, again consistent with their higher FGF-2 staining. We noted that BB94 did not reduce the FGF-2 levels in the Chloe explant, possibly due to the presence of MMP-resistant aggrecan in this setting. Collectively, these data demonstrate that a TIMP loss promotes FGF-2 signaling, which in turn suppresses the IHH pathway, triggering aberrant cartilage homeostasis and growth plate integrity, ultimately resulting in abnormal postnatal bone development and body posture, as summarized in Fig. 10 D.

## Discussion

Whole-body removal of the entire TIMP gene family has directly demonstrated their redundancy and identified their indispensable role in skeletal growth, stature, and lifespan. Adult quadruple TIMP-deficient mice have widespread chondrodysplasia throughout the appendicular and axial skeleton and epiphyseal growth plate closure of long bones, a phenomenon underlying growth retardation. Pectus excavatum is an extreme manifestation of the TIMP-deficient sternum and, to our knowledge, reported in only one other mouse GEMM model, of *Ptpn11* loss in the mesenchymal lineage (Lapinski et al., 2013). Pectus deformities affect up to 0.3% of the human population in association with a range of connective tissue disorders including Marfan syndrome and related disorders (Tocchioni et al., 2013). Our combinatorial TIMP knockouts also reveal the exclusive requirement of TIMP2 plus TIMP3 for embryonic survival, although their deletions do not impose remarkable skeletal deformities in the rare surviving animal. Further deletion of *Timp1* and *Timp4* greatly improves embryonic survival, although these mice are runted and have a severely shortened lifespan. Surprisingly, a single *Timp*3 allele is sufficient for a near-normal lifespan, as shown by QT3^+/−^ mice, yet these mice display pervasive bone abnormalities. Overall, TIMP-deficient GEMMs offer new models to study pectus excavatum, bone elongation, and chondrocyte biology. Furthermore, these combinatorial GEMMs help reveal the redundancy of TIMP activity in select systems such as the chondrocytes in growth plate cartilage and the mammary stem and progenitor pools in the mammary epithelium (Jackson et al., 2015).

The human growth plate undergoes progressive narrowing, ultimately disappearing with the cessation of growth upon sexual maturity, although the complex mechanisms governing this process are not completely understood (Staines et al., 2018). Recent studies emphasized a role of matrix remodeling in this process as active matrix fragments are shed, and endochondral bone formation is also influenced by epigenetic regulation of several metalloproteases (Carpio et al., 2016; Coghlan et al., 2017). Several MMPs (e.g., MMP-2, -3, -8, -9, -10, -12, and -13) and ADAMTSs (e.g., ADAMTS1, 4, and 5) enzymes are present in the growth plate, and null mutations in some MMPs (MMP-9, -13, and -14) affect growth plate morphology (Gack et al., 1995; Mattot et al., 1995; Hou et al., 2004; Little et al., 2005). Growth plates also express TIMPs in chondrocyte zones: *Timp1* in all zones, *Timp2* in proliferating and hypertrophic zones, and *Timp3* in the hypertrophic zone (Takahashi et al., 2005). Axial and appendicular QT growth plates had intense metalloprotease activity. The unleashed metalloproteases resulted in closure of both femoral and tibial growth plates through bone bridges, although a lower ratio of femur:tibia length indicated a greater sensitivity of the femur growth plate to metalloprotease degradation.

A single *Timp3* allele in QT3^+/−^ mice significantly rescued the long bone shortening, consistent with TIMP3 having the broadest metalloprotease inhibition profile. The intact growth plate in *Timp3*^−/−^ mice shows that its absence alone is insufficient to initiate growth plate disruption. Likewise, the individual loss of TIMP2 or TIMP4, and their combined absence with TIMP3 (*Timp*2^−/−^*Timp3*^−/−^; *Timp*2^−/−^*Timp3*^−/−^*Timp4*^−/−^), presented a normal growth plate, whereas the additive TIMP1 loss (*Timp*1^−/−^*Timp3*^−/−^*; Timp*1^−/−^*Timp2*^−/−^*Timp3*^−/−^) led precipitously to bone bridges and complete penetrance of the growth plate closure phenotype. Thus, TIMP1, along with TIMP3, is required for normal growth plate development during postnatal to pubertal lifespan. TIMP1 is a weak inhibitor of MT-MMPs but efficiently blocks most other MMPs, whereas TIMP3 is a robust ADAMTS inhibitor (Murphy, 2011). Interestingly, pronounced bone phenotypes in TIMPless and combinatorial TIMP-deficient mice also point to the redundancy among TIMP functions. Appearance of weak or no phenotypes in single gene knockouts of most MMPs have often raised speculation about functional redundancy among these matrix remodeling enzymes, which has been confirmed through the complete deletion of TIMPs.

MMP and ADAMTS members degrade a wide range of cartilage matrix components including aggrecan and their cleaved neopeptides can be detected in humans and rodents suggesting an active role of these metalloproteases in aggrecan turnover (Lark et al., 1997; van Meurs et al., 1999; Makihira et al., 2003). As reported, a baseline level of aggrecan turnover is mediated by MMPs, whereas ADAMTSs are known to initiate cartilage damage in arthritis. Previously, aggrecan turnover by MMPs was thought to be a dispensable mechanism in growth plate biology, as it was studied in WT background where growth plate disruption was not apparent (Little et al., 2005). When we compared aggrecan degradation by MMPs versus ADAMTSs in mice lacking most TIMPs, i.e., QT3^+/−^, Jaffa (ADAMTS resistant) and Chloe (MMP resistant) aggrecan knock-in mutations produced distinct outcomes on cartilage development in appendicular and axial skeleton. MMP-resistant aggrecan rescued chondrocyte/FGF2 organization, growth plate integrity, and bone length in both femur and tibia of C-QT3^+/−^ mice, while only the tibial cartilage was rescued in the ADAMTS-resistant J-QT3^+/−^ mice. This demonstrates that TIMP regulation of MMP activity is necessary for normal femur and tibia growth plate, and that the femoral growth plate is clearly more susceptible to excess MMP activity than the tibial growth plate. The lack of rescue in the femur of J-QT3^+/−^ mice argues that at least some ADAMTS activity is necessary for normal femoral growth plate development. Similarly, exacerbation of sternum shortening in the C-QT3^+/−^ mouse points to MMP cleavage at aggrecan N^341^/F^342^ as necessary for normal sternum chondrocyte development. Overall, these two mutations in aggrecan affect the axial skeleton differently, since MMP resistance exacerbates rather than rescues the QT3^+/−^ shortened sternum, and ADAMTS resistance had no effect. Therefore, simultaneous regulation of two major metalloprotease classes, MMP and ADAMTS, by the TIMP gene family is crucial to processes that determine optimal bone growth and isometry in the mouse.

At the molecular level, our study uncovers the TIMP-metalloprotease axis as an overarching control on FGF-2 bioavailability, which subsequently controls *Ihh* expression during the chondrocyte maturation program. TIMP-deficient GEMMs phenocopy numerous traits in mice with increased FGF signaling such as dwarfism, shortened long bones, macrocephaly, and reduced bone mass (Xiao et al., 2004; Sobue et al., 2005). Mice with activating FGFR3 mutations have a smaller body size, kinky tails, dorsal kyphosis, a dome-shaped skull, shorter long bones encompassing disorganized chondrocyte columns in growth plates, shortening of the nasal and frontal bones of the skull, misaligned closure of the upper/lower incisors, and delayed formation of secondary ossification centers (Wang et al., 1999), traits seen in the quadruple TIMP knockouts. Further, the epiphyseal growth plates of mice with FGFR3 mutations have smaller proliferating and hypertrophic zones (Wang et al., 1999; Lee et al., 2017) similar to TIMPless mice. Humans with FGFR3 gain-of-function point mutations also present a variety of skeletal dysplasias (Lee et al., 2017). In a pharmacological model, dosing with an IHH pathway inhibitor led to a shortened femur and premature growth plate closure (Kimura et al., 2008), similar to our TIMP-deficient mice. Collectively, the strong similarities in phenotypes among these GEMMs strengthen FGF-2 and IHH as core cartilage development pathways dependent on the natural metalloprotease inhibitor activity. Given the complexity of the skeletal system, which also heavily relies on balanced osteoblast and osteoclast activity, we have recently reported low bone mass in TIMP-deficient long bones due to higher Rankl activity in osteoblasts, which is also downstream of FGFR3 signaling (Su et al., 2010; Wen et al., 2016; Chen et al., 2019).

In summary, this comprehensive study of combinatorial TIMP-deficient GEMMs uses global expression profiling, biochemical and in situ analyses, and rationalized aggrecan-resistant knock-in GEMMs to uncover metalloprotease regulation of FGF-2 as a crucial event in the chondrocyte maturation program, underlying correct growth plate development and bone elongation responsible for attaining proper body stature.

## Materials and methods

### Key resources

Key resources are listed in Table S2.

### Mice

All mice used in this study are of pure C57BL/6 background. Mice were housed and cared for in accordance with the guidelines approved by the Canadian Council for Animal Care and the Animal Care Committee of the Princess Margaret Cancer Centre (Toronto, Canada). We used individual TIMP knockout mice that were previously generated using homologous recombination; T1^−/−^ mice have a stop codon within each reading frame of the third exon of *Timp1* gene (Soloway et al., 1996); T2^−/−^ mice are devoid of the first exon of *Timp2* and additional 5′ genomic sequences (Wang et al., 2000); T3^−/−^ mice lack 6 kb from exons 2 and 3 of *Timp3*, including translation initiation sequence and sequences encoding amino acids important for TIMP inhibitory activity (Leco et al., 2001); and T4^−/−^ mice have deletion of a 2.4-kb genomic fragment containing exons 1–3 of *Timp4*, including translation initiation codon (Koskivirta et al., 2010; Fig. 1 A). These mice were bred to produce different combinations of whole-body TIMP knockout GEMMs, and with Chloe and Jaffa knock-in mice (Little et al., 2005, 2007). In Jaffa knock-in mice, a mutation was inserted in exon 7 of aggrecan gene (*Acan*) to change the amino acid sequence from 374ALGS to 374NVYS, eliminating aggrecanase cleavage site 373E/374A, whereas in the Chloe knock-in strategy, exon 7 of *Acan* was mutated to change the amino acid sequence from 342FFG to 342GTR, disrupting the MMP cleavage site 341N/342F. These knock-in strains in C57BL/6 background were bred through several crosses of combinatorial TIMP-deficient mice to produce Chloe-QT3^+/−^ (C-QT3^+/−^) and Jaffa-QT3^+/−^ (J-QT3^+/−^) GEMMs.

### Radiography

Formalin-fixed bones were imaged using a Faxitron MX-20 digital x-ray system with a 24kV, 4-s exposure time for 2D analysis. For whole-body micro-CT imaging, formalin-fixed mouse skeletons were placed in a GE Locus Ultra Micro-CT (GE Medical Systems) and subjected to a 16-s Anatomical Scan Protocol (total of 680 images) at 80 kV, 70 mA, using a 0.15-mm Cu Filter, to achieve ∼150-µm resolution. The same machine was also used for live-mouse imaging at acquisition parameters 80 kV, 50 mA; 16-s anatomical scan; 154-µm isotropic voxels (total of 680 slices) and 3D rendered using Siemens Inveon. For high-resolution micro-CT imaging, fixed mouse legs were immobilized on 1.25% agarose. Specimens were scanned in 360° rotation using a Siemens Inveon Micro-CT high-resolution scanner (Siemens Medical Systems) with the x-ray source at 80 kVp and 0.5 μA. 3D micro-CT data were reconstructed at 13.5-µm resolution. Raw data processing was performed using ImageJ software (National Institutes of Health), and 3D isosurfaces were rendered using Microview software (GE Healthcare). Bone length was measured digitally with ImageJ.

### Histology

Dissected bones from mice aged ≥2 wk were formalin fixed for 48 h at RT, or 8-wk-old bones were fixed for 72 h at 4°C before decalcification in 14% EDTA (Sigma-Aldrich). Embryonic and newborn bones were fixed in 4% paraformaldehyde for 24 h at 4°C. All tissues were embedded in paraffin, sectioned at 4 µm, placed on Superfrost slides, and stained with hematoxylin and eosin (H&E). Unstained sections were deparaffinized and hydrated with water for further staining. Safranin O staining was performed as described previously (Mahmoodi et al., 2005) with a few modifications. Briefly, slides were stained with Weigert’s iron hematoxylin (BDH), 0.001% fast green solution (BDH), 1% acetic acid, and 0.1% Safranin O (Thermo Fisher Scientific). Stained specimens were photographed using an Infinity2 camera (Lumenera) and Olympus SZ2-1LST dissecting microscope (uEye software; 4×/0.5-NA objective) or Zeiss Axiolab microscope (Zen blue software; 20×/0.5-NA or 2.5×/0.075-NA objective) at RT.

### Histomorphometry

To establish the relative proportion of tissues in the sternum and spine, H&E-stained sections were examined with a Merz eyepiece graticule (Merz and Schenk, 1970), and the cross-sectional areas of cartilage, bone matrix, and bone marrow were measured and expressed as percentages of total area examined.

### Skeletal staining

The skin and soft tissues were removed from embryos or adult mice before fixation in 95% EtOH. Mice were stained for 2 d in 0.1 mg/ml alcian blue (Sigma-Aldrich) in an 80%:20% volumetric solution of EtOH:glacial acetic acid, and then rehydrated in sequential EtOH solutions of 70% (twice), 40%, and 15% and distilled H_2_O. Skeletons were cleared overnight in 1% KOH (Sigma-Aldrich) and stained for 2 d in 0.01 mg/ml alizarin red (Sigma-Aldrich) in 1% KOH. Stained specimens were photographed using an Infinity2 camera (Lumenera) and Olympus SZ2-1LST dissecting microscope.

### Microarray

Flash-frozen sternums from 4-wk-old WT and QT mice were thawed in RNAlater-ice per the manufacturer’s instructions (Ambion). Sternal cartilage pads were dissected and homogenized in 800 µl of Trizol (Invitrogen). RNA was extracted as described previously for cartilage (Ali and Alman, 2012), with two additional ethanol wash steps. Quality was assessed using the Agilent 2100 Bioanalyzer, and RNA integrity for all samples was between 8.3 and 9.2. For microarray analysis, 200 ng of each sample was loaded onto Mouse Gene ST 2.0 chips (Affymetrix). Raw CEL files were preprocessed using the standard robust multiarray average algorithm built in Partek Genomics Suite. Statistical analysis was conducted using the Limma package (v3.10.3) of the Bioconductor open-source software project (Gentleman et al., 2004; Smyth, 2004) in R statistical environment (v 2.14.2); linear modeling was applied to identify genes altered in QT sternal cartilage relative to WT samples. Bayesian moderation of the standard error implemented was conducted on all model-based *t* tests (Smyth, 2004). In addition, multiple testing was corrected by a false discovery rate adjustment (Storey and Tibshirani, 2003). Significant differential expressed genes between WT and QT were identified based on a false discovery rate cutoff of <0.05. Pathway enrichment analysis was performed using the Enrichr gene list enrichment analysis tool (Chen et al., 2013). Further, gene ontology functional enrichment analysis was performed using g:Profiler, and an enrichment map was generated using cystoscope (v3.6.0) with a q value cutoff of 0.01. Enriched pathways were identified using AutoAnnotate (Kucera et al., 2016).

### RT-PCR

Whole sternums and proximal tibia heads of mice were cleaned of connective tissue, snap frozen immediately following dissection, and kept at −80°C. Frozen bones were pulverized, and total RNA was extracted using TRIzol reagent (Invitrogen; 15596-026). For cultured chondrocytes, RNA was extracted after 3 d of culture from duplicate cultures per mouse using 400 µl of TRIzol reagent per well of a 24-well plate. Results from duplicate wells were averaged and normalized to DMSO-treated control samples from the same mouse. 1 μg of RNA was reversed-transcribed to cDNA using qScript cDNA SuperMix (Quanta Biosciences). Quantitative real-time PCR was performed using the TaqMan Gene Expression Master Mix (Life Technologies) for sternum samples and using PerfeCTa SYBR Green SuperMix with ROX (Quanta Biosciences) for primary chondrocytes. PCR products were analyzed using ABI PRISM 7900HT Sequence Detections System (Applied Biosystems). For TaqMan analysis, the following primers from Applied Biosystems were used: B-actin (4352933E), aggrecan (Mm00545794_m1), Collagen2a1 (Mm01309565_m1), IHH (Mm00439613_m1), and Collagen10a1 (Mm00487041_m1). Primers used for RT-PCR analysis are listed in Table S3.

### 217 Probe Synthesis

^1^H-217 and ^3^H-217 were obtained from the same precursor, using a Universal NovaTag resin (Novabiochem) through fluorenylmethyloxycarbonyl chloride (Fmoc) solid phase peptide synthesis. Fmoc groups were removed by consecutive treatment of the resin with 20% piperidine (Fluka) in anhydrous *N*,*N*-dimethylformamide (DMF; Fluka) for 10 and 7 min. Unless mentioned otherwise, all numbers of equivalents of reagents are given relative to the resin loading (mmol · g^−1^). A fivefold excess of Fmoc amino acids (Novabiochem) was preactivated for 5 min using COMU (Novabiochem; 4.75-fold equivalent) and diisopropylethylamine (10-fold equivalent) in DMF. This mixture was added to the resin for coupling (25 min). Capping of unreacted amino groups as well as acetylation of N-termini were performed using *N*-acetyl imidazole (Sigma-Aldrich) in DMF (0.3 M) for 20 min. Incorporation of the phosphinic block (1.2-fold excess) was achieved using *N*,*N*′-diisopropylcarbodiimide (Sigma-Aldrich)/ClHOBt (Molekula) in DMF (1.2-fold excess) with an overnight coupling. The selective removal of trityl groups on the commercial resin was performed using 0.6 M HOBt in tetrafluoroethylene/CH_2_Cl_2_ (1:1). The liberated free amino function was subsequently coupled to BocNH-Peg27-COOH (Novabiochem) with the help of COMU. Cleavage of the pseudopeptides from the solid support was performed by treating the resin several times with a solution of trifluoroacetic acid:H_2_O:triisopropylsilane (95%:2.5%:2.5%; Sigma-Aldrich) to generate, after reverse-phase HPLC purification, the pure ^1^H-217 and ^3^H-217 probe precursor bearing a unique free amine.

### Imaging

For radioactive imaging, a mixture of ^1^H-217 and ^3^H-217 was injected intravenously (150 µCi/ml PBS for total 0.7 mM; 10 mg/kg into 12-wk-old mice). Mice were anesthetized, and body temperature was maintained at 38°C for 1 h before sacrifice. Mice were immersed in dry ice/isopentane to prevent tissue redistribution, and whole-body sections (20 µm) were prepared at −20°C with a slicing microtome (Leica Microsystems). Sagittal sections were desiccated (24 h, RT) before radioimaging (β-imager TM 2000, Biospace Lab). For fluorescence imaging, 4-wk-old mice were injected with MMPSense750 Fast (intravenously, 0.08 nmol/g; PerkinElmer) and sacrificed 7 h later. Organs were extracted and imaged (Maestro system; Cambridge Research and Instrumentation). Multispectral image cubes containing multiple 10-nm bandwidth fluorescence emission signals were acquired at identical exposure times and spectrally unmixed using the fluorescence emission spectra from the injected compound.

### Neonatal chondrocyte culture

Neonatal chondrocyte cultures were derived by adapting described protocols (Gosset et al., 2008). Neonatal mouse pups (3 d old) were sacrificed and dissected to collect the rib cage. Rib cages were digested with serum-free αMEM and 2.5 mg/ml collagenase A for 20 min at 37°C, with shaking at 200 rpm, and then with serum-free DMEM and 2.5 mg/ml collagenase A for 4 h at 37°C. The tissue digest was passed through a 70-µm strainer, and single cells were seeded in complete culture medium (αMEM, 10% FBS, 10 mM β-glycerophosphate, and 50 µg/ml ascorbic acid; 10^5^ cells/24-well plate) with medium changed every 2 d. Inhibitors used were BB94, 10 µM (Sigma-Aldrich); TAPI-1, 20 µM (Peptides International); and cyclopamine, 20 µM (Selleckchem). To assess proteoglycan production, cells were fixed (10 min, 1:7:2 solution of 37% formalin: EtOH:distilled H_2_O), stained overnight (0.1 mg/ml alcian blue [Sigma-Aldrich] in 4:1 EtOH:glacial acetic acid), and photographed using an Infinity2 camera (Lumenera) mounted on an Olympus CKX41 microscope (Fig. S5 C).

### Femoral distal head explant culture

3-wk-old mice were dissected to collect femurs, and after complete removal of muscles, the femoral distal head was carefully separated from the rest of long bone using forceps under sterile conditions. The femoral head was then transferred to a 48-well plate (as shown in Fig. 10 C) and cultured in 300 µl serum-free DMEM for 72 h (5% CO_2_, 37°C) with BB94 (10 µM) or vehicle control DMSO, as done elsewhere for femoral distal head explant culture (Stanton et al., 2011). The inhibitor was added every 24 h, and culture supernatant was collected for ELISA.

### FGF-2 ELISA

FGF-2 ELISA was performed using the commercially available kit EMFGF2 (Thermo Fisher Scientific) and following the manual. Culture supernatant and standard were added (100 µl) to the well of a precoated 96-well ELISA plate and incubated overnight at 4°C with gentle shaking. After washing with provided wash buffer, 100 µl of biotinylated antibody was added to the wells and incubated at RT for 1 h. Streptavidin-HRP antibody was added after washing and incubated for 45 min at RT. After washing, plates were developed using tetramethylbenzidine substrate and incubating at RT for 30 min. Absorbance was read at 450 nm using a POLARstar Omega microplate reader (BMG Labtech). FGF-2 concentration was estimated using the standard curve.

### Immunostaining

4-µm wax sections were deparaffinized, hydrated, and preincubated with 10% goat serum in PBS for 1 h at RT. Sections were incubated overnight at 4°C with primary antibodies (FGF-2, Abcam; ab8880 at 1:100 dilution; perlecan, Invitrogen; MA5-14641 at 1:100 dilution; and aggrecan, Abcam; ab3778 at 1:150) in 10% goat serum followed by a wash and incubation with their corresponding secondary antibodies conjugated with Alexa Fluor 488, 594, and 647 (Life Technologies; 1;100 dilution) for 1 h at RT. Images were captured with a Zeiss LSM700 confocal microscope using Zen blue software. A 20×/0.8-NA objective was used, with air medium at RT.

### Statistical analysis

Prism software (GraphPad) was used for all analyses. Data distributions were assumed to be normal. Unpaired Student’s *t* test, Wilcoxon matched-pair test, and one-way ANOVA tests were used for pairwise and multiple comparisons, respectively. One-way ANOVA with Sidak’s, Dunnett’s, and Bonferroni’s post hoc multiple comparison tests were performed for multiple comparison. The χ^2^ test was used to determine whether offspring of a given genotype were observed in a Mendelian ratio. The χ^2^ test for independence was used to compare mouse survival rates between genotypes. All graphs are plotted as mean with error bars representing SEM; *, P < 0.05; **, P < 0.01; ***, P < 0.001; ****, P < 0.0001. Statistical analyses performed on microarray and mass spectrometry datasets are detailed in their respective sections.

### Data and software availability

Microarray data generated in this study was submitted to Gene Expression Omnibus under accession no. GSE60451.

### Online supplemental material

In Fig. S1, rib head enlargement at both costovertebral and costotransverse joints of QT mice are shown. In Fig. S2, minor defects in axial and appendicular bone of QT embryos are displayed. In Fig. S3, growth plate organization and chondrocyte maturation zone are shown spanning days 2 to 28 in QT and WT, as well as tibia and femur lengths of individual TIMP knockouts. In Fig. S4, shortening of sternebrae and enlargement of cartilaginous joint of sternum are shown across several GEMMs. In Fig. S5, up-regulated and down-regulated pathways are summarized from microarray analyses of sternal cartilage, along with MMP activity in skeletal system and *Timp* gene expression in primary chondrocytes across experimental and control mouse cohorts. Table S1 lists genes significantly altered in TIMPless sternal cartilage, Table S2 lists key resources, and Table S3 lists primers used for SYBR Green analysis.

## Supplementary Material

Supplemental Materials (PDF)

Table S1 (Excel)
