# Supplementary material for: Metalloprotease inhibitor TIMP proteins control FGF-2 bioavailability and regulate skeletal growth
Source: J Cell Biol. 2019 Aug 1;218(9):3134–52. doi: 10.1083/jcb.201906059 (PMC6719459; doi:10.1083/jcb.201906059)
Supplement: Supplemental Materials (PDF) [file JCB_201906059_sm.pdf]

Supplemental material

Saw et al., https://doi.org/10.1083/jcb.201906059

Provided online is one table in Excel. Table S1 lists genes significantly altered in TIMPless sternal cartilage.

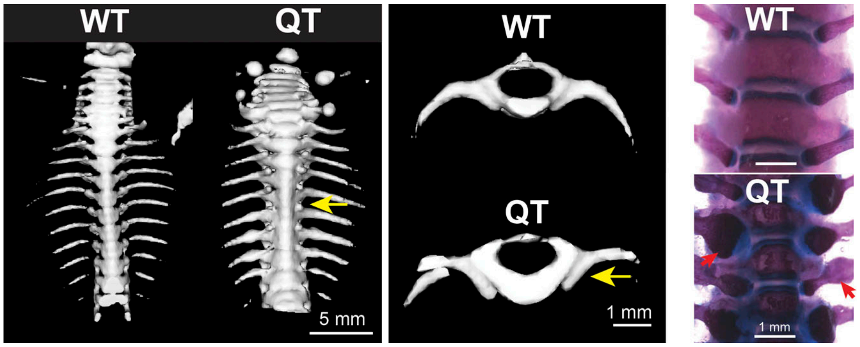

Figure S1. **Bulging of QT ribs at the costovertebral/costotransverse joints.** Faxitron x-ray images of the spine and ribs of 4-wk-old mice. Images show bony enlargements (yellow arrow) of rib in QT mice; coronal (left) and transverse plane (middle). Alcian blue and alizarin red staining showing rib head enlargement (red arrow) at both costovertebral and costotransverse joints in QT mice (right).

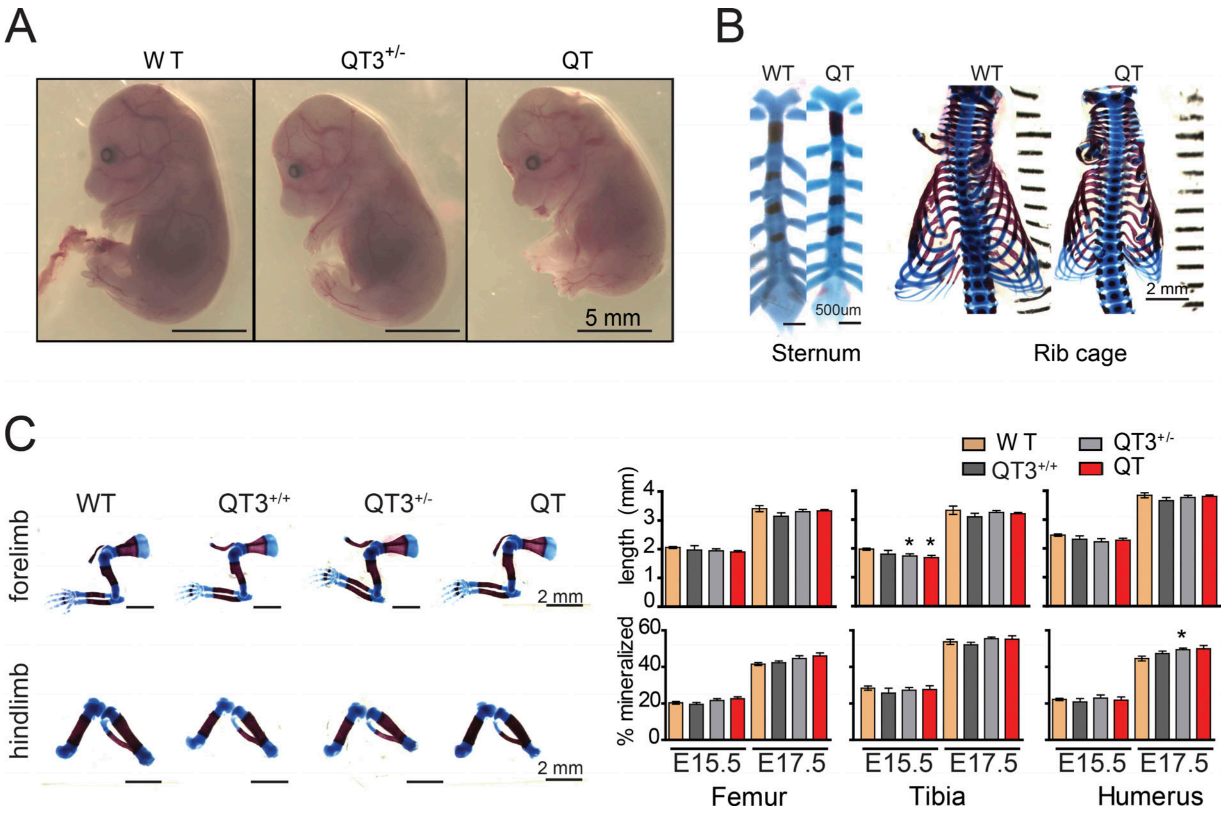

Figure S2. **Embryonic skeletal assessment. (A)** E17.5 WT, QT3$^{+/-}$, and QT embryo (E17.5) display minor differences. **(B)** Alcian blue– and alizarin red–stained axial skeletons, sternum, and rib cage of WT and QT mice at E15.5. **(C)** Alcian blue– and alizarin red–stained appendicular bones. Alizarin red indicates degree of mineralization in bone (bottom histogram). Appendicular bone length (top histogram) at E15.5 and E17.5 (WT, *n* = 8–14; QT3$^{+/+}$, *n* = 4–13; QT3$^{+/-}$, *n* = 9–14; QT, *n* = 4–5). Mean values of each dataset are plotted with error bars representing SEM. Data were compared using one-way ANOVA following Dunnett's multiple comparison, *, P < 0.05 compared with WT.

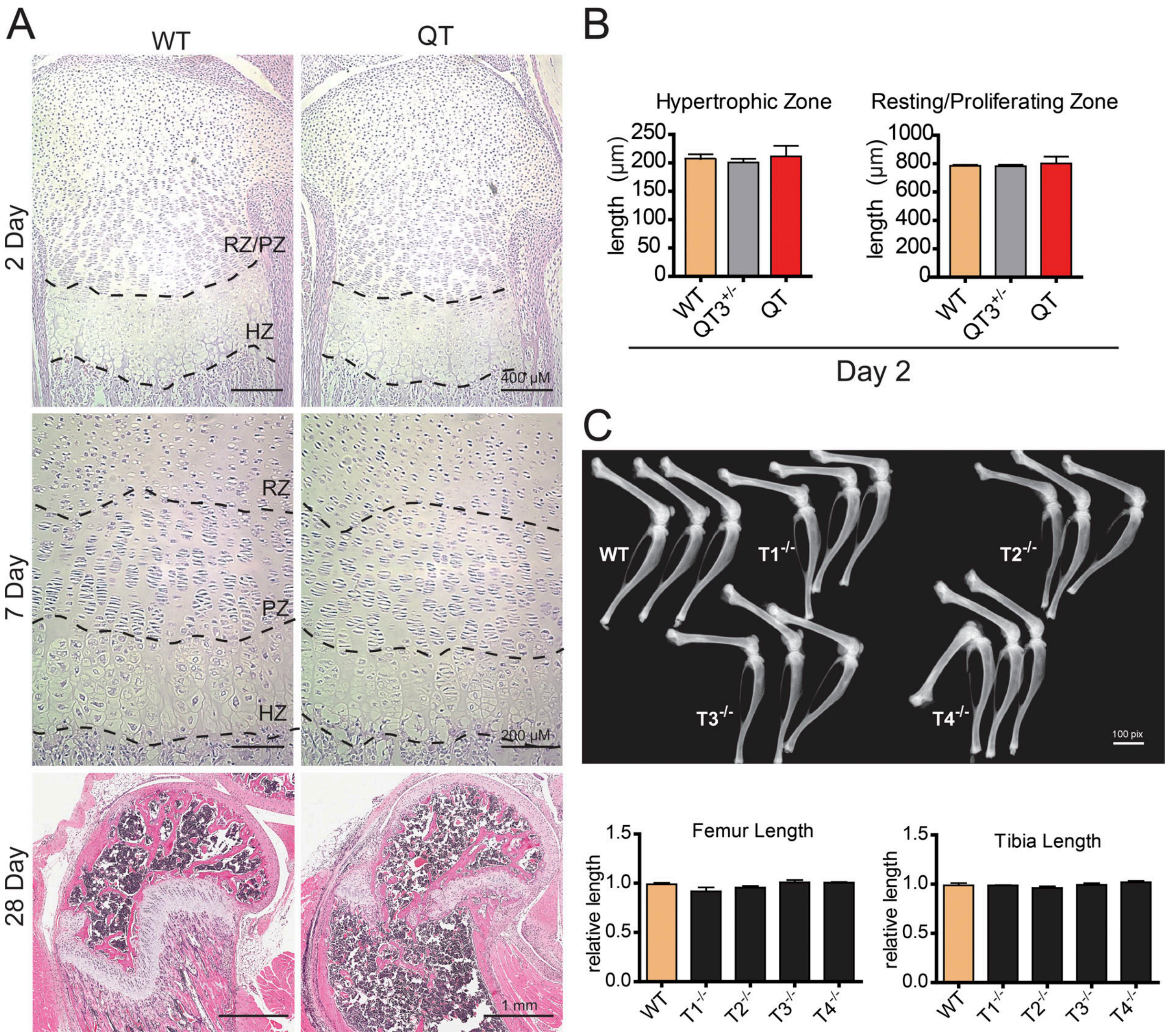

Figure S3. **Postnatal growth plate and long bone assessment. (A)** Images of tibia (day 2; top panel) and femur (day 7, middle panel; day 28, bottom panel) of mice. Black dotted line marks the zones of chondrocyte maturation in GP. Thickness of morphologically delineated resting/proliferating zones (RZ/PZ) and hypertrophic zone (HZ). **(B)** Quantitative analysis of developmental zones of GP of 2-d-old WT, QT3$^{+/-}$, and QT mouse knee joint, indicating no major abnormality in 2-d-old TIMP-deficient mice. WT, *n* = 5; QT3$^{+/-}$, *n* = 5; and QT, *n* = 3. Mean values of each dataset are plotted with error bars representing SEM. Data were compared using Bonferroni's multiple comparison test. **(C)** Faxitron images of leg bone of WT mice or mice lacking a single TIMP, as indicated. Quantitative data (lower panel) of femur and tibia length of individual TIMP knockouts indicate that loss of a single TIMP is not sufficient to elicit bone shortening (*n* = 3 each group). Mean values of each dataset are plotted with error bars representing SEM. Data were compared using Bonferroni's multiple comparison test.

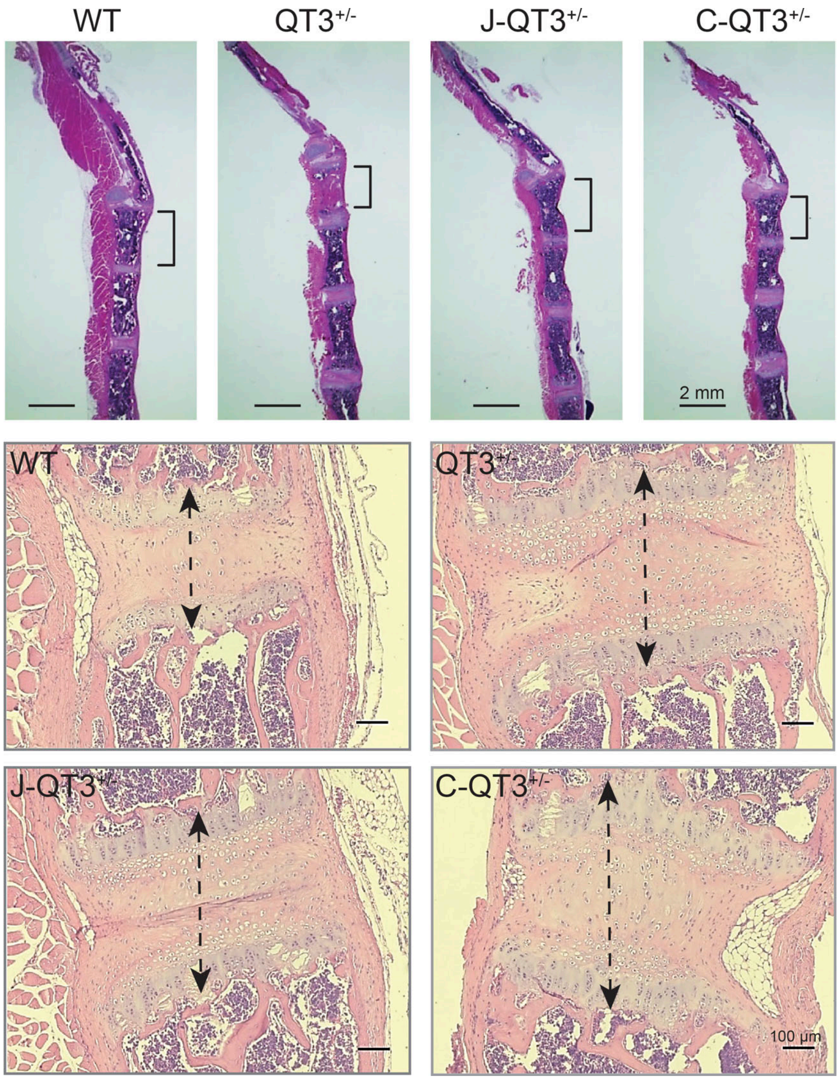

Figure S4. **Sternum shortening in Chloe-QT3$^{+/-}$ mice.** H&E-stained sections of sternum from 8-wk-old WT, QT3$^{+/-}$, J-QT3$^{+/-}$, and C-QT3$^{+/-}$ mice. Comparison of sternebrae (black brackets in the upper panel) and cartilaginous joints bordered by two growth plates (black dashed lines with arrowheads in the lower panel).

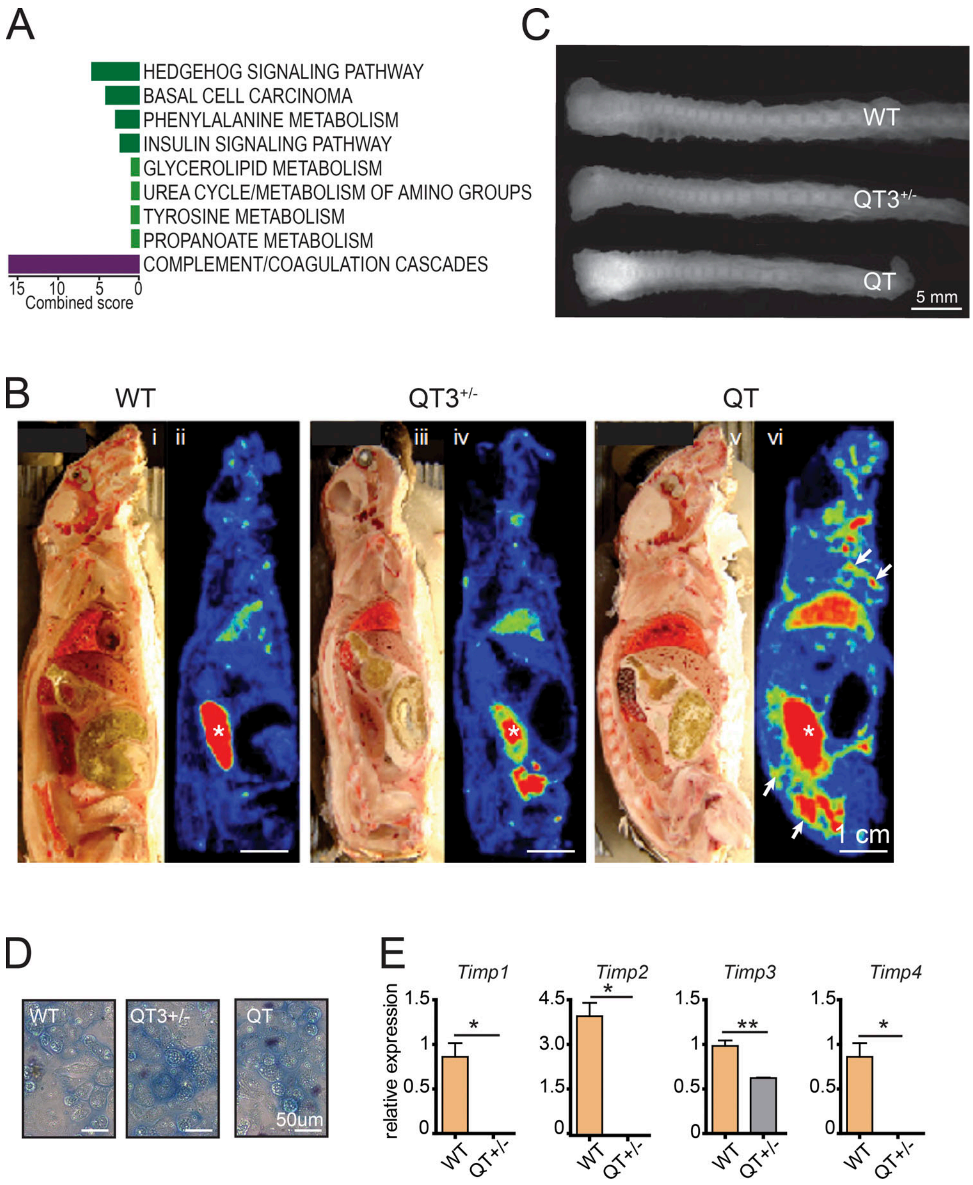

Figure S5. **Imaging of metalloprotease activity. (A)** Pathway enrichment using Enrichr showing up-regulated (purple) and down-regulated (blue) pathways in TIMPless sternal cartilage with a combined score ≥1. **(B)** Sagittal sections from 12-wk-old mice injected with a radioactive probe ^{3}H-217. Bright-field anatomic images (i, iii, and v) and corresponding radioactive signal images (ii, iv, and vi) show MMP activity (blue = low activity, red = high activity). White arrow indicates higher activity in bone, and white asterisk indicates nonspecific signal in kidney due to urinary excretion. **(C)** Ex vivo spines from 4-wk-old mice showing MMPSense750 fluorescent signal (white). A more intense signal indicates higher MMP activity. **(D)** Alcian blue staining of proteoglycan production in 3-d-old primary chondrocyte cultures show functional chondrocytes. **(E)** *Timp* (-1, -2, -3, and -4) gene expression in primary chondrocytes (WT, n = 3; QT3$^{+/-}$, n = 3). Mean values of each dataset are plotted with error bars representing SEM. Datasets were compared by unpaired t test: *, P < 0.05; **, P < 0.01.

Table S2. **Key resources**

Reagent or resource	Source	Identifier
**Antibodies**		
FGF-2	Abcam	ab8880
Perlecan	Invitrogen	MA5-14641
Goat anti-rabbit	Life Technologies	A21245
Goat anti-mouse	Life Technologies	A11001
Aggrecan	Abcam	ab3778
**Chemicals, peptides, recombinant proteins**		
TRIzol reagent	Life Technologies	15596018
qScript cDNA SuperMix	Quanta Biosciences	84035
TaqMan Gene Expression Master Mix	Applied Biosystems	4369016
PerfeCTa SYBR Green SuperMix with ROX	Quanta Biosciences	95055-02K
BB94	Sigma-Aldrich	SML0041
TAPI-1	Peptides International	NH-3855-PI
Cyclopamine	Selleckchem	S1146
AZD4547	Selleckchem	S2801
**Commercial assays**		
Mouse Gene ST 2.0 chips	Affymetrix	902119
FGF-2 ELISA	Thermo Fisher Scientific	EMFGF-2
**Experimental models: mouse/cell/explant**		
C57BL/6 mice	NA	NA
Chondrocyte primary cell culture (mouse)	NA	NA
Femoral distal head explant culture (mouse)	NA	NA
**Software and algorithms**		
Limma package (v3.10.3) of Bioconductor	Open source	NA
R statistical environment (v 2.14.2)	Open source	NA
g:Profiler	Reimand et al., 2007	NA
Enrichr	Chen et al., 2013	NA
Enrichment map	Merico et al., 2010	NA
AutoAnnotate	Kucera et al., 2016	NA
ImageJ	Open source, National Institutes of Health	NA

NA, not applicable.

Table S3. **Primers used for SYBR Green analysis**

Gene	Forward primer sequence (5′ to 3′)	Reverse primer sequence (5′ to 3′)
Agg	GGCTTTCCTGCAGAAGTCAC	CTGTGCCTCCTCAAATGTCA
Boc	ACAAGAGGACACACACCACG	TCACACTCTCGCACCTCGTA
Cdo	CCAGACCACCCTGAGGATAA	TGGAATGCATTAATTGTCGG
Col2a1	GCAAGATGAGGGCTTCCATA	CTACGGTGTCAGGGCCAG
ColXa1	ACCAGGAATGCCTTGTTCTC	CATAAAGGGCCCACTTGCTA
Cxcl12	AGGGCACAGTTTGGAGTG	GCGCTCTGCATCAGTGAC
Cxcr4	ACTCCTTCATCCTTTTGGGG	TCTGCTCATGGAGTTGAGTG
Fgf2	GTCACGGAAATACTCCAGTTGGT	CCCGTTTTGGATCCGAGTTT
Gapdh	GGGCATCTTGGGCTACACTGAG	CTCTTGCTCAGTGTCCTTGCTGG
Gli1	CTTGTGGTGGAGTCATTGGA	GAGGTTGGGATGAAGAAGCA
Gli2	CGAGTGCAACGTTTCCACAG	GTCAGGTTGCTGTCTACGCT
Hhip	CTTGTAATTGGGATGGAATGC	TCAAGGAGCCTTACTTGGACA
Ihh	TGACAGAGATGGCCAGTGAG	CAATCCCGACATCATCTTCA
Inhba	GATCATCACCTTTGCCGAGT	TGGTCCTGGTTCTGTTAGCC
Mmp3	CAGACTTGTCCCGTTTCCAT	GGTGCTGACTGCATCAAAGA
Mmp13	GGTCCTTGGAGTGATCCAGA	TGATGAAACCTGGACAAGCA
Mmp19	GCAGCTGTGGCTGGCATTCT	GCCTGAAGTCATCAGCTCCTT
Pdpn	CACCTCAGCAACCTCAGAC	AAGACGCCAACTATGATTCCAA
Ptch1	AATTCTCGACTCACTCGTCCA	CTCCTCATATTTGGGGCCTT
Sfrp5	GGAGCACATCTGTTCCATGA	CTGGACAACGACCTCTGCAT
Timp1	AGGTGGTCTCGTTGATTTCT	GTAAGGCCTGTAGCTGTGCC
Timp2	GAATCCTCTTGATGGGGTTG	CGTTTTGCAATGCAGACGTA
Timp3	TAGACCAGAGTGCCAAAGGG	CCAGGATGCCTTCTGCAAC
Timp4	GGAGGGGCTGTTTGTTACGA	TCTTTGCTGGCAGGGACTAC
Tnfaip6	ATACAAGCTCACCTACGCCGAA	ATCCATCCAGCAGCCAGACAT

References

Chen, E.Y., C.M. Tan, Y. Kou, Q. Duan, Z. Wang, G.V. Meirelles, N.R. Clark, and A. Ma'ayan. 2013. Enrichr: interactive and collaborative HTML5 gene list enrichment analysis tool. *BMC Bioinformatics*. 14:128. https://doi.org/10.1186/1471-2105-14-128

Kucera, M., R. Isserlin, A. Arkhangorodsky, and G.D. Bader. 2016. AutoAnnotate: A Cytoscape app for summarizing networks with semantic annotations. *F1000 Res.* 5:1717. https://doi.org/10.12688/f1000research.9090.1

Merico, D., R. Isserlin, O. Stueker, A. Emili, and G.D. Bader. 2010. Enrichment map: a network-based method for gene-set enrichment visualization and interpretation. *PLoS One.* 5:e13984. https://doi.org/10.1371/journal.pone.0013984

Reimand, J., M. Kull, H. Peterson, J. Hansen, and J. Vilo. 2007. g:Profiler--a web-based toolset for functional profiling of gene lists from large-scale experiments. *Nucleic Acids Res.* 35:W193–W200. https://doi.org/10.1093/nar/gkm226
